# Double Attack to Oxidative Stress in Neurodegenerative Disorders: MAO-B and Nrf2 as Elected Targets

**DOI:** 10.3390/molecules28217424

**Published:** 2023-11-04

**Authors:** Filippo Basagni, Maria Luisa Di Paolo, Giorgio Cozza, Lisa Dalla Via, Francesca Fagiani, Cristina Lanni, Michela Rosini, Anna Minarini

**Affiliations:** 1 Department of Pharmacy and Biotechnology, Alma Mater Studiorum-University of Bologna, Via Belmeloro 6, 40126 Bologna, Italy; filippo.basagni2@unibo.it; 2 Department of Molecular Medicine, University of Padova, Via G. Colombo 3, 35131 Padova, Italy; marialuisa.dipaolo@unipd.it (M.L.D.P.); giorgio.cozza@unipd.it (G.C.); 3 Department of Pharmaceutical and Pharmacological Sciences, University of Padova, Via F. Marzolo 5, 35131 Padova, Italy; lisa.dallavia@unipd.it; 4 Consorzio Interuniversitario Nazionale per la Scienza e Tecnologia dei Materiali (INSTM), 50121 Firenze, Italy; 5 Department of Drug Sciences (Pharmacology Section), University of Pavia, V.le Taramelli 14, 27100 Pavia, Italy; fagiani.francesca@hsr.it (F.F.); cristina.lanni@unipv.it (C.L.); 6 Division of Neuroscience, IRCCS San Raffaele Hospital, Via Olgettina 60, 20132 Milan, Italy

**Keywords:** pioglitazone, MAO-B, Nrf2, electrophile, cinnamic acid, oxidative stress

## Abstract

Oxidative stress and neuroinflammation play a pivotal role in triggering the neurodegenerative pathological cascades which characterize neurodegenerative disorders, such as Alzheimer’s and Parkinson’s diseases. In search for potential efficient treatments for these pathologies, that are still considered unmet medical needs, we started from the promising properties of the antidiabetic drug pioglitazone, which has been repositioned as an MAO-B inhibitor, characterized by promising neuroprotective properties. Herein, with the aim to broaden its neuroprotective profile, we tried to enrich pioglitazone with direct and indirect antioxidant properties by hanging polyphenolic and electrophilic features that are able to trigger Nrf2 pathway and the resulting cytoprotective genes’ transcription, as well as serve as radical scavengers. After a preliminary screening on MAO-B inhibitory properties, caffeic acid derivative **2** emerged as the best inhibitor for potency and selectivity over MAO-A, characterized by a reversible mechanism of inhibition. Furthermore, the same compound proved to activate Nrf2 pathway by potently increasing Nrf2 nuclear translocation and strongly reducing ROS content, both in physiological and stressed conditions. Although further biological investigations are required to fully clarify its neuroprotective properties, we were able to endow the pioglitazone scaffold with potent antioxidant properties, representing the starting point for potential future pioglitazone-based therapeutics for neurodegenerative disorders.

## 1. Introduction

Pioglitazone is an insulin sensitizer structurally belonging to the thiazolidinedione family that has been approved since 1999 for the treatment of type-2 diabetes. It acts as an agonist of peroxisome proliferator-activated receptor γ (PPAR-γ), a PPAR’s isoform mainly present in adipose tissue and macrophages. PPAR-γ activation also plays a pivotal role in inflammatory response, attenuating the release of proinflammatory cytokines and inhibiting NF-κB signalling, whereas at central level, counteracts neuroinflammation through the modulation of protein misfolding [1,2]. Due to the spreading of neuroinflammation in neurodegenerative conditions and its ability to penetrate the blood–brain barrier (BBB) in therapeutic concentrations [3], pioglitazone has been evaluated as therapeutic tool to tackle neurodegeneration. It exerted promising neuroprotective effects in Parkinson’s disease (PD) animal models, showing a reduction in glial activation and preventing dopaminergic neuronal loss [4]. These pioglitazone in vivo properties proved to be related more to cellular mechanisms involving monoamine oxidase B (MAO-B) inhibition rather than PPAR-γ-mediated anti-inflammatory effects [5]. MAO-B, a member of the mono amine oxidase family, is a flavin-dependent mitochondrial enzyme, which catalyses the oxidative deamination of amine-containing neurotransmitters to produce ammonia, aldehydes and hydrogen peroxide (a reactive oxygen species—ROS). Interestingly, an over-expression and over-activity of MAO-B was found in the central nervous system (CNS) of elderly people affected by neurological disorders, thus contributing to neurodegenerative oxidative damage [6,7,8].

In vitro studies revealed pioglitazone, the best among other glitazones, as a selective submicromolar inhibitor of *h*MAO-B. The high-resolution crystal structure of *h*MAO-B with pioglitazone showed no covalent engagement, differently from most common marketed MAO-B inhibitors, highlighting a competitive mechanism of inhibition [9]. This repositioning as an MAO-B inhibitor, combined with the already known PPAR-γ agonism, and corroborated by its clinical safety since its marketing as a drug, pushed pioglitazone into several preclinical and clinical trials for the treatment of neurodegenerative disorders [10]. Unfortunately, all clinical trials involving pioglitazone to modify the progression of Alzheimer’s disease (AD) and PD registered almost no beneficial effects, thus highlighting the need for more effective treatments [11,12,13]. On this basis, thanks to its antioxidant–anti-inflammatory properties at a central level, we herein sought to explore the possibility to combine the beneficial effects of pioglitazone deriving from MAO-B inhibition to the induction of cytoprotective genes’ transcription, with the aim to possibly prevent and reduce oxidative damage with a multi-layered strategy.

The nuclear factor erythroid 2-related factor 2 (Nrf2) is a transcription factor that plays a pivotal role in the inducible cell defence system by coordinating a multifaced response to various forms of stress and inflammatory processes. The interaction between Nrf2 and its repressor Kelch-like ECH-associated protein 1 (Keap1) is a crucial point for regulating Nrf2 function. The electrophilic modulation of Keap1 at critical cysteines represents the most common way for physiological and pharmacological Nrf2 induction. The chance to specifically activate inducible antioxidant defence via precise electrophilic addition emerges as a valuable strategy to tackle neurodegeneration [14], thus increasing the interest in identifying Nrf2 inducers as therapeutic agents, with dimethyl fumarate representing a notable success story [15]. Moreover, several dietary polyphenols and their polyphenolic derivatives are well recognized to exert remarkable neuroprotective properties through the activation of Nrf2 pathway, besides a direct antioxidant activity [16,17]. Their activity was mainly ascribed to the presence of (pro)electrophilic features, which can trap Keap1 at the cytosolic level and trigger cytoprotective responses [18,19].

A structural analysis on pioglitazone scaffold underlined the thiazolidinedione ring as the driving motif in MAO-B interaction, whereas different substituents at the entrance of the binding site were well tolerated [20]. Thus, with the aim to empower the neuroprotective efficacy of pioglitazone with the Nrf2-driven antioxidant response, we maintained the 5-(4-hydroxybenzyl)thiazolidine-2,4-dione moiety of pioglitazone, and exploited the hydroxyl function as the attaching point for introducing electrophilic warheads with probed Nrf2-inducing capacities.

**Figure 1:**
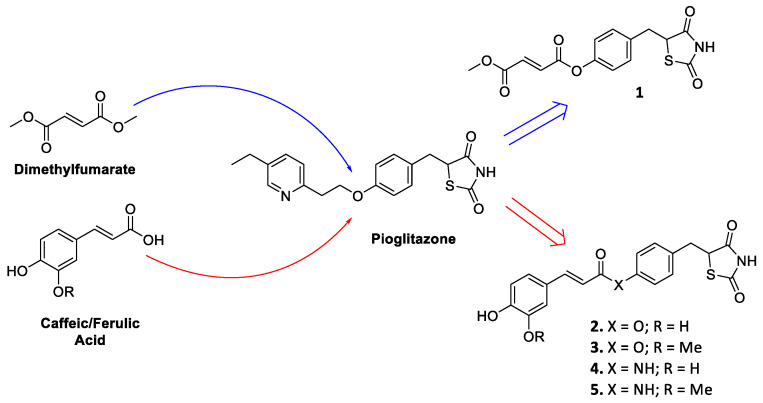
Drug design of conjugates **1**–**5**.

Firstly, as a proof of concept, we linked the most known Nrf2 inducer monomethyl fumarate, the active metabolite of the drug dimethyl fumarate [21,22], to the identified pioglitazone pharmacophoric head by means of ester functionality, resulting in compound **1** (Figure 1). Furthermore, caffeic and ferulic acids, carrying the characterizing electrophilic features of hydroxycinnamic core, were selected as other Nrf2-inducing molecular appendages and were exploited with the same conjugating approach, affording compounds **2** and **3** (Figure 1). For cinnamic derivatives, an alternative coupling method was also followed, leading to the corresponding amides **4** and **5** (Figure 1), as this modification is generally associated to an increased chemical stability. As the racemization of pioglitazone occurs reasonably rapidly [23], which precludes the administration of either enantiomer to enhance therapeutic specificity as well as a more thorough analysis of their differences in binding affinities, the new compounds were synthesized and assayed in their racemic form.

## 2. Results and Discussion

### 2.1. Chemistry

**Scheme 1:**
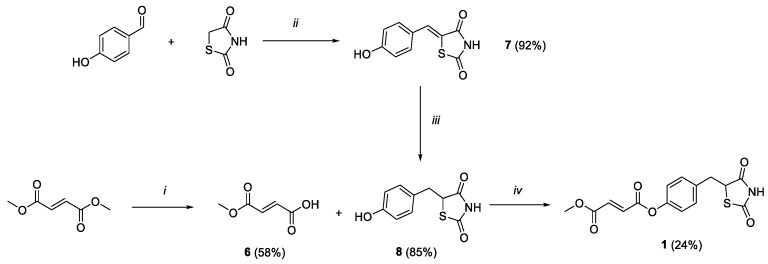
Reagents and conditions: (*i*) LiOH 1N, acetone, rt, 2h; (*ii*) benzoic acid, piperidine, toluene, reflux, 6h; (*iii*) DMG/CoCl_2_/DMF, NaBH_4_, MeOH/NaOH 1M/H_2_O, rt, 2h; (*iv*) HOBt, EDC, Et_3_N, DCM, 0 °C-rt, 36 h.

**Scheme 2:**
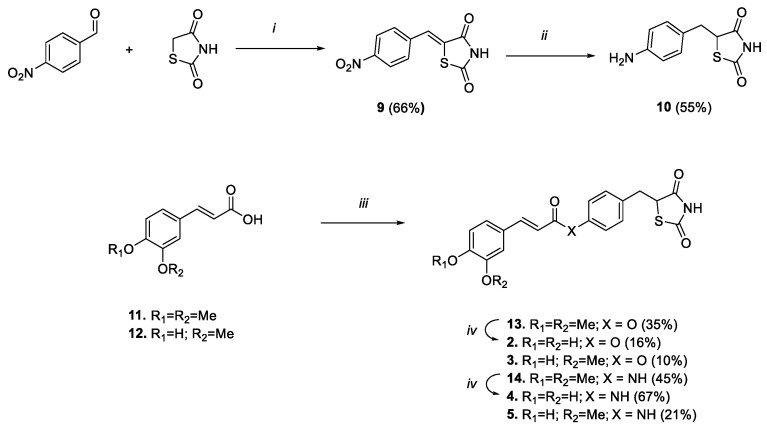
Reagents and conditions: (*i*) benzoic acid, piperidine, toluene, reflux, 6 h; (*ii*) DMG/CoCl_2_/DMF, NaBH_4_, MeOH/NaOH 1M/H_2_O, rt, 2 h; (*iii*) **8** or **10**, HOBt, EDC, Et_3_N, DMF, 0 °C-rt, 48 h; (*iv*) BBr_3_ 1M in DCM, DCM, 0 °C-rt, 1 h.

Compounds **1**–**5** were synthesized as reported in Scheme 1 and Scheme 2.

The synthetic strategy exploited to obtain compound **1** is outlined in Scheme 1. Firstly, monomethylfumarate (**6**) was achieved by modifying a reported ester hydrolysis under basic conditions [24]. Key intermediate **8** was obtained within a two-step process. 4-hydroxybenzaldehyde and thiazolidine-2,4-dione underwent a Knoevenagel condensation to result in the benzylidene intermediate **7**. The double bond was reduced by applying a previously reported procedure, where a complex of cobalt (II) chloride and dimethylglyoxime was exploited to potentiate a reducing efficacy of sodium borohydride, thus easily discarding the hydride ion to reduce the highly conjugated unsaturation [25]. Finally, compound **1** was obtained from a coupling reaction between **6** and intermediate **8,** using HOBt ad EDC as activating agents and adding triethylamine as base to promote a coupling adduct formation.

To obtain the amido analogues (Scheme 2), the aniline derivative **10** was prepared by following the same two-step condensation–reduction procedure that was previously followed to gain the phenolic analogue **8**, albeit double the amount of reducing agent and complex was needed to reduce in a single step both the benzylidene double bond and the nitro group. Thus, the carboxylic function of the selected acids (i.e., ferulic or 3,4-dimethoxy cinnamic) was subsequently activated using HOBt and EDC, and coupled with phenol **8** or aniline **10**, to result in compounds **13**, **14**, **3** and **5** (Scheme 2). Lastly, 3,4-dimethoxy derivatives **13** and **14** were converted into catechol analogues **2** and **4**, respectively, by unmasking 3,4-dimethoxy functions using a boron tribromide-mediated demethylation procedure.

^1^H NMR spectra indicate that compounds **2**–**5**, featuring a carbon−carbon double bond between the catechol ring and the carbonyl function, have an *E* configuration as revealed by the large spin coupling constants (around 15–16 Hz) of *α*-H and *β*-H on double bonds. Furthermore, deepened NMR studies were conducted to confirm the predicted and intended structure of compounds (see Supplementary Information for details).

### 2.2. Effects and Kinetic Characterization of Compounds **1**–**5** on hMAOs

To evaluate the potential efficacy of hybrids **1–5** as human MAO inhibitors, they were tested in vitro versus both the recombinant *h*MAO-B and *h*MAO-A isoforms. Table 1 reports the inhibition constant values (K_i_) of the tested compounds **1–5** and that of pioglitazone, used as MAO-B reference compound, and caffeic acid phenethyl ester (CAPE) used as reference for Nrf2 activity [17,26,27]. Upon stating the importance of counteracting *h*MAO-B activity in neurodegenerative disorders, selectivity was also calculated and reported.

From the obtained data, it clearly emerges that the linkage of the thiazolidinedione head of pioglitazone to fumaric, caffeic and ferulic fragments to provide the hybrid compounds **1, 2** and **3**, respectively, allowed for the maintenance of the capability to inhibit MAO, with *h*MAO-B preference, even if with lower potency than pioglitazone, which was confirmed to act as a selective and competitive inhibitor of *h*MAO-B [9]. All compounds proved to inhibit *h*MAO-B with low micromolar efficacy (K_i_) and notable selectivity, whereby K_i_ for the *h*MAO-A isoform was higher than 20 µM for all the tested hybrids. Compound **2** was found to be the most potent *h*MAO-B inhibitor among the hybrid compounds, albeit less potent than pioglitazone (K_i_ values 530 vs. 61 nM, respectively). Thus, the ferulic and caffeic appendages did not compromise the capability of the pioglitazone moiety to inhibit MAO activity, even if their free acids were found to be not active on MAOs up to a 100 μM concentration. Differently, CAPE showed the capability to inhibit *h*MAO-B with a K_i_ value (1.8 μM) of magnitude comparable to that of other derivatives, except for the caffeic analogue **2**, where the potency and selectivity were significantly improved (i.e., about four times higher and seven times higher than CAPE, respectively). This was not a surprise, since other caffeic/ferulic derivatives have already emerged as MAO inhibitors [28,29]. The fumaric moiety of compound **1** allowed in maintaining the micromolar selective inhibition of *h*MAO-B, even with a decrease in inhibitory potency of 50 times in comparison with pioglitazone. Notably, the substitution of the ester linker with the amide one, performed on most active compounds **2** and **3**, decreased the inhibitory capability of the compounds by a factor of four–five (**2** vs. **4**: K_i_ 0.5 vs. 2.5 µM; **3** vs. **5**: K_i_ 0.99 vs. 3.3 µM). To corroborate the solidity of the obtained results, the chemical stability of derivative **2** was evaluated at the same experimental conditions exploited for determining *h*MAO-B activity by means of UHPLC-MS experiments. After 1.5 h (maximal time of *h*MAO activity assay), besides pure compound elution, no detection of the respective acid was observed, corroborating the chemical stability of the ester function in the used experimental conditions (Figure S2).

**Figure 2:**
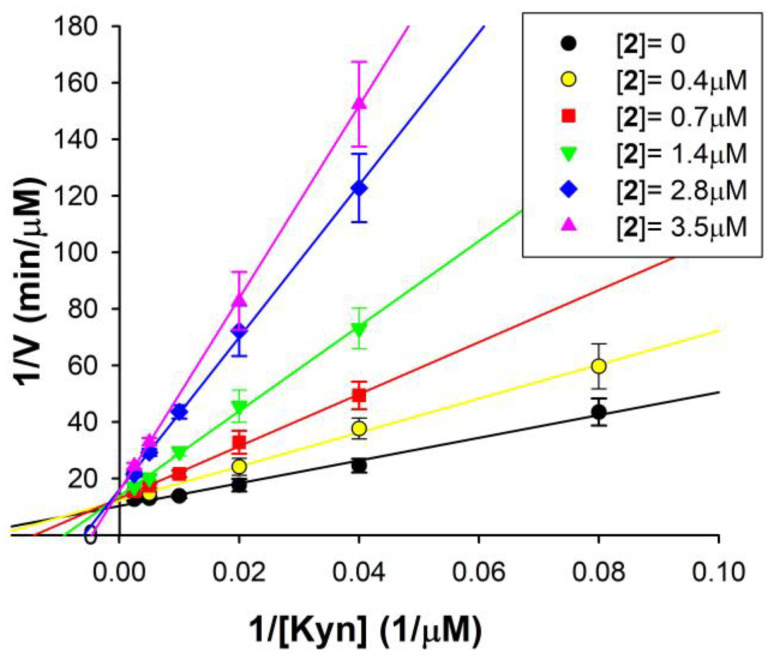
Mixed inhibition of *h*MAO-B by compound **2**. Double reciprocal plots of *h*MAO-B activity in the absence (●) and in the presence of different **2** concentrations (0.4–3.5 µM). The *K_EI_* = 0.45 ± 0.08 µM and *K_EIS_* = 11.25 ± 4.12 µM for the mixed mode of inhibition of **2** were calculated via global fit analysis (GraphPad Prism 9.0 software). Continuous lines are the result of linear regression analysis of plotted data (r^2^ > 0.98).

For the most potent and selective *h*MAO-B inhibitor of the series, compound **2**, the mechanism of inhibition was investigated. Figure 2 shows the double reciprocal plots of the rate of reaction of *h*MAO-B at different substrate concentrations, in the presence of various **2** concentrations. These plots clearly show that both K_m_ (the intercept on the x-axis is −1/K_m_) and V_max_ (intercept on the y-axis is 1/V_max_) are affected by the presence of the inhibitor. In detail, the apparent K_m_ value increases and the apparent V_max_ decreases with increasing **2** concentrations. This behaviour reveals a mixed inhibition mechanism, as confirmed via the global fit analysis of the experimental data carried out by applying the software GraphPad Prism 9.0 (mixed inhibition model presented the best fit with r^2^ > 0.98).

By this analysis, the following inhibition constant values of compound **2** for *h*MAO-B were calculated: *K_EI_* = 0.45 ± 0.8 µM (for the free enzyme), in good agreement with the K_i_ value reported in Table 1, and *K_EIS_* = 11.25 ± 4.12 µM (for the enzyme substrate complex). Thus, the K_i_ values reported in Table 1 (performed at [S] = 10 µM < K_m_) are only the dissociation constant values of the free enzyme–inhibitor complex (*K_EI_*), whereas for observing the effect on the ES–I complex (depending on the *K_EIS_*), saturating substrate concentrations and high inhibitor concentrations should have been used. Indeed, it should be noted that the K_EI_ is more than one order of magnitude lower than *K_EIS_*, suggesting that **2** has a much better affinity for the free enzyme than for the enzyme substrate complex. It means that the preferred **2** binding site is inside the *h*MAO-B active site, where it binds in competition with the substrate. Additionally, this binding is reversible, because no inactivation and no time-dependent inhibition was found for **2** vs. *h*MAO-B.

This result confirms that the exploited conjugation approach did not alter, excluding a reasonable loss in potency and selectivity, the promising MAO-B inhibitory profile of pioglitazone, whose selectivity and reversibility emerged as a promising source of therapeutics for neurodegeneration, avoiding the adverse effect of gold-standard irreversible MAO-B inhibition (i.e., cheese effect) and the onset of compensatory mechanism while maintaining beneficial outputs [30]. This road ahead is also supported by the approval of the highly selective and reversible MAO-B inhibitor safinamide for the treatment of PD [31].

### 2.3. Docking Analysis of Compounds **2**–**5** against hMAO-B

In order to rationalize the interaction of the new hybrids against *h*MAO-B, a series of molecular docking experiments were carried out on more selective compounds **2**–**5**. Albeit the molecules have been biologically investigated in the racemic form, as previously specified, they were herein studied in silico with the *R*-configuration. An X-ray crystallographic study conformation of *R*-pioglitazone in complex with *h*MAO-B (PDB code: 4A79) is available, and, on the basis of model fitting to electron density, this enantiomer appears to preferentially bind to the enzyme [9].

**Figure 3:**
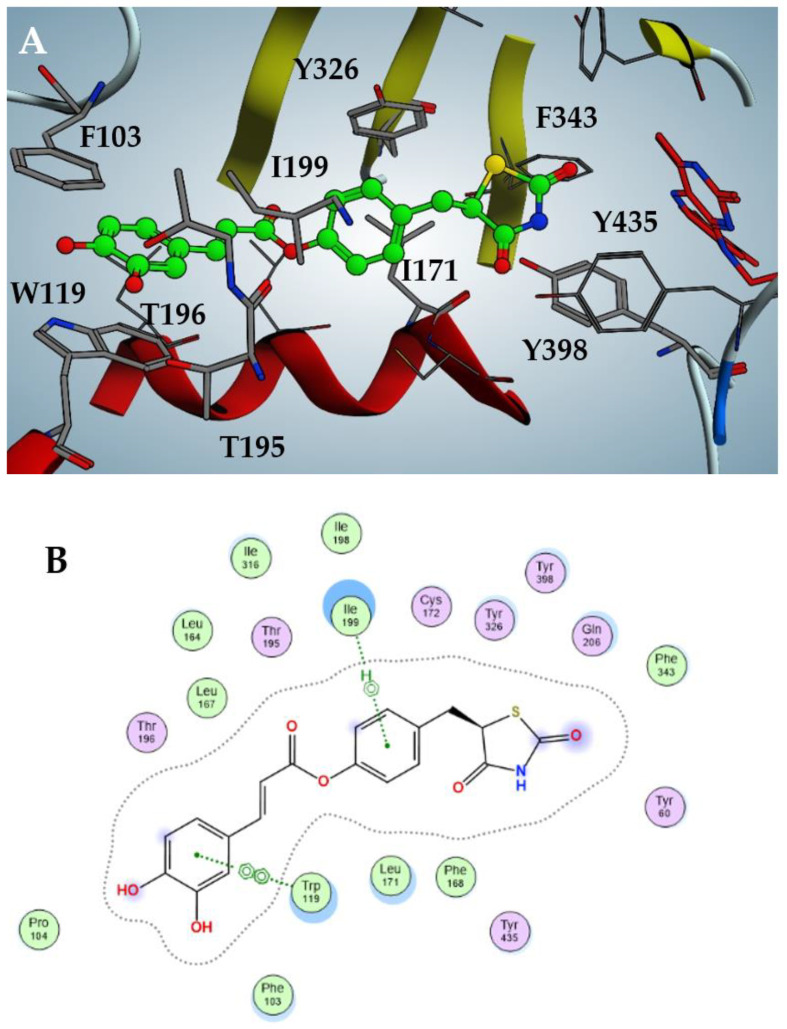
Docking pose of compound **2** against *h*MAO-B (**A**) with highlighted interactions involved (**B**).

In particular, as shown in Figure 3, the best inhibitor **2** fits inside the catalytic pocket of *h*MAO-B, almost occupying the entire volume. The thiazolidinedione moiety protrudes, facing the FAD flavin ring, stabilized by hydrophobic interactions with Phe343, Tyr398 and Tyr435; the phenol core is sandwiched between apolar Ile171 and Ile199, accompanied by a T-shaped pi-stacking with Tyr326. The catechol moiety is projected towards the entrance of the catalytic pocket and establishes efficient π–π stacking with Phe103 and Trp119. Finally, weak hydrogen bonds between the *meta*-hydroxy function and Thr195/196 should be noted.

**Figure 4:**
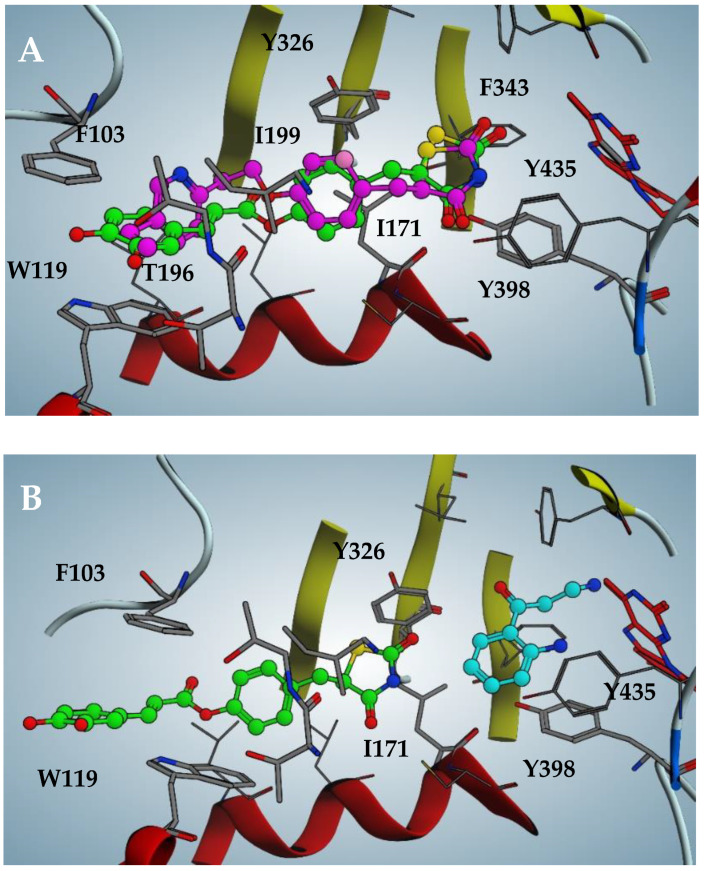
(**A**) Docking pose of compound **2** compared to pioglitazone pose (violet in figure, PDB code: 4A79) against *h*MAO-B. (**B**) Docking pose of **2** when substrate kynuramine (cyan in figure) is present within the active site of *h*MAO-B.

The pose likeliness of **2**, obtained through in silico analysis, is confirmed by the good overlap with the X-ray crystallographic conformation of the pioglitazone in complex with *h*MAO-B (violet in figure, PDB code: 4A79; Figure 4A). Intriguingly, **2** is able to interact with the *h*MAO-B pocket even in the presence of the substrate kynuramine, previously docked into the catalytic site (cyan in the figure, Figure 4B). However, in this case, the inhibitor is forced to retreat towards the entrance of the catalytic tunnel by 2.5 Å, losing most of the efficient apolar interactions discussed previously. This results in a loss of affinity of two orders of magnitude of the estimated binding affinity, expressed as a dissociate constant (pKd = 5.0), which perfectly fits with the biochemical analysis of the mechanism of action of **2**, mixed-competitive at high inhibitor concentrations.

Furthermore, the methoxy group of ferulic derivative **3** does not involve any appreciable difference in the in vitro activity, a result also confirmed by the docking analysis (pKd = 6.8), where the methoxy group settles easily in an apolar region.

Amido derivatives **4** and **5** poses (Figures S3 and S4) are comparable to their ester analogues, albeit the amide group presents an evident distortion due to the van der Waals contact with Phe168, reflecting a loss of affinity of almost one order of magnitude when comparing **4** with **2**.

### 2.4. Cell Viability

**Figure 5:**
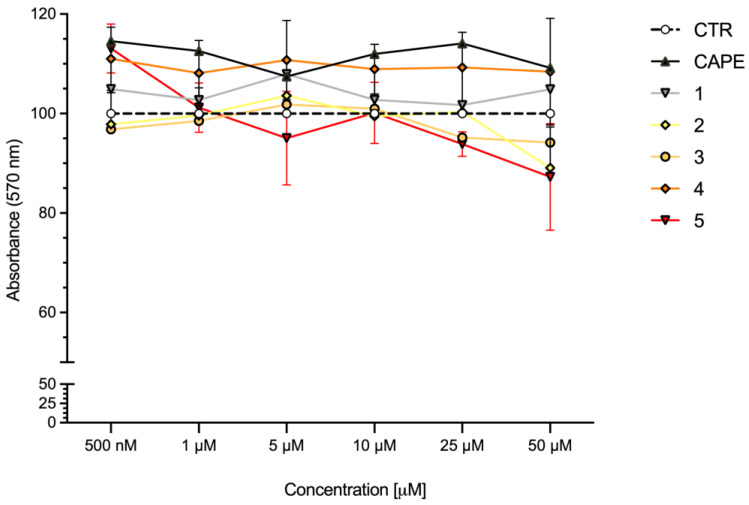
Cell viability in SH-SY5Y cells exposed to CAPE and compounds **1**–**5**. SH-SY5Y cells were treated with CAPE, and compounds **1**–**5** at the indicated concentrations for 24 h. Cell viability was assessed via MTT assay. Data are expressed as means of percentage of cell viability. Two-way ANOVA, multiple comparison test; only CAPE vs. **5** was statistically significant (*p* < 0.05); n = 4.

To define the concentration range to use in the in vitro experimental settings, the cytotoxicity of compounds **1**–**5** was evaluated in SH-SY5Y human neuroblastoma cells, in comparison with CAPE, the reference compound for Nrf2 activation [26,27] (Figure 5). Cells were treated with compounds at concentrations ranging from 0.5 to 50 μM for 24 h and cell viability was evaluated via MTT assay. All compounds were well tolerated at each concentration, resulting in no or very low reduction in cell viability.

### 2.5. Nrf2 Nuclear Translocation

**Figure 6:**
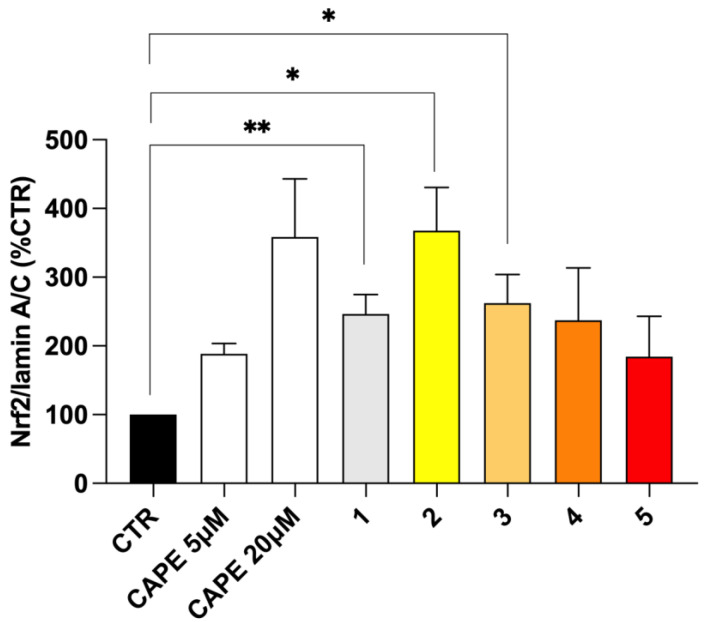
Nrf2 nuclear translocation in SH-SY5Y cells treated with compounds **1**–**5**. SH-SY5Y cells were treated with CAPE 5 µM and 20 µM as reference and compounds **1**–**5** at the concentration of 5 μM for 3 h. After isolation, nuclear extracts were examined via Western blot analysis and Nrf2 expression was determined using an anti-Nrf2 antibody. Anti-lamin A/C was used as protein loading control. Results are shown as Nrf2/Lamin A/C ratio (%CTR) ± SEM. * *p* < 0.05, ** *p* < 0.01 vs. control, Bonferroni’s multiple comparison test.

In physiological conditions, the repressor Keap1 traps Nrf2 at the cytosolic level, preventing the activation of Nrf2 pathway. The disruption of Keap1–Nrf2 interaction allows for Nrf2 nuclear translocation, thus resulting in the transcriptional activation of its target cytoprotective genes. Hence, Keap1 acts as a redox sensor, which can be functionally modified by both reactive endogenous species under oxidative/electrophilic stress and exogenous pharmacologic tools [19,32]. We and others have previously developed several compounds bearing caffeic and ferulic fragments able to activate Nrf2 in terms of the up-regulation of Nrf2 expression, translocation into the nucleus and induction of the Nrf2-dependent defensive genes, with (pro)electrophilic features emerging as key players [33,34,35,36]. Herein, to analyse the effect of the new molecules on the Nrf2 downstream cascade, the ability of compounds **1**–**5** to increase Nrf2 nuclear translocation was preliminary evaluated as a pivotal step in Nrf2 signalling activation (Figure 6). Based on MAO-B inhibitory potency and cell toxicity assay, compounds were studied in SH-SY5Y human neuroblastoma cells at the concentration of 5 μM. Given the high structural analogy with our derivatives, we selected the well-known Nrf2 inducer CAPE [37,38] as the reference compound, and verified its ability to induce Nrf2 nuclear translocation at different concentrations, ranging from 5 µM to 50 µM in our experimental model (Figure S6). Nrf2 nuclear content was assessed via Western blot analysis. In our experimental conditions, pioglitazone was not able to induce Nrf2 nuclear translocations, until concentrations higher than 50 µM. Consistently, data from literature highlight discrepancies about pioglitazone-mediated Nrf2 activation, indicating its ability to activate Nrf2 in longer timeframes and different in vitro and in vivo experimental conditions [39,40]. Based on these results, and as suggested for rosiglitazone [41], we might speculate that the pioglitazone-induced Nrf2 modulation mainly relies on an indirect effect through the modulation of PPAR-γ/Nrf2 axis, thanks to its PPAR-γ agonist properties, thus requiring longer time sets to activate the signalling cascade instead of a direct activity on the Nrf2 pathway. As shown in Figure 6, all tested compounds, as well as CAPE 5 µM and 20 µM, induced Nrf2 nuclear translocation, albeit with different extents. In particular, only ester derivatives **1**–**3** significantly resulted in Nrf2 translocation, with caffeic derivative **2** emerging as the most active. The preliminary docking performed on human Keap-1 confirms the excellent positioning of compound 2 within the BTB domain pocket, where cysteine 151 is located (see Figure S5). This result is consistent with a covalent mechanism of action that involves the thiol-reactive electrophiles of compound **2** and CAPE, although the allocation of the latter is notably less efficient, as supported by the in vitro data (see Figure S5). Conversely, the amide analogues **4** and **5** promoted Nrf2 translocation at a lower extent. Furthermore, such as that which occurred with MAO activities, even in this case, the minimal structural discrepancies occurring between amide **4** and **5** and esters **2** and **3** resulted in prominent differences in biological activities. Particularly, all these new molecules hold the characteristic (pro)electrophilic fragments, which however, in this case, resulted to be insufficient to activate Nrf2, as reported for other ferulic and caffeic derivatives [35,36]. In this regard, optimal target interaction might play a major role, but further biological investigations are needed to fully clarify the mechanism of Nrf2 activation and the effect on the downstream expression of cytoprotective genes.

### 2.6. Antioxidant Efficacy in HepG2 Cells

As hydroxycinnamic acids are well-known natural compounds endowed with antioxidant properties [42], researchers evaluated whether the caffeic and ferulic acid moieties, combined with the 5-(4-hydroxybenzyl)thiazolidine-2,4-dione moiety of pioglitazone, enriched the latter’s biological profile with regard to a direct antioxidant activity. Thus, on the basis of the *h*MAO-B inhibitory potency and on the Nrf2 nuclear translocation results, the most promising compounds **2** and **3** were chosen for the evaluation of their antioxidant activity in the human hepatic HepG2 cells, a valuable cellular system to assess antioxidant properties of the compounds [43,44]. CAPE and pioglitazone were also tested as reference compounds. HepG2 cells were pre-loaded with dichlorofluorescein diacetate (DCF-DA), a fluorescent probe to detect ROS generation. Then, the tested compounds (**2**, **3**, pioglitazone and CAPE, 5 µM final concentration) were added to the PBS–glucose medium. Immediately, antimycin A (10 µM), an inhibitor of the complex III of the mitochondrial electron transport chain, was also added as an ROS inducer. The oxidation rate of DCF, that is, the generation rate of ROS, was detected for 30 min, and for each sample, the corresponding control was also run.

**Figure 7:**
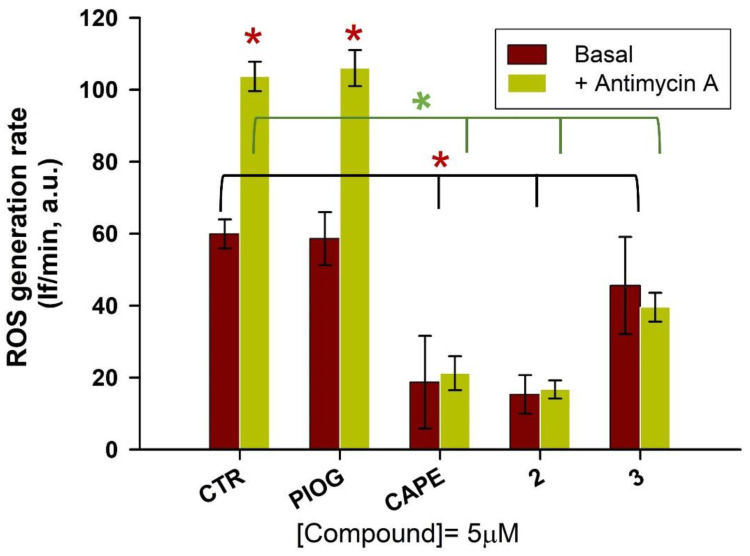
Evaluation of the effect compounds **2** and **3** on the ROS production induced by antimycin A (10 µM) and on the basal ROS production in HepG2 cells. CAPE and pioglitazone were also tested as reference compounds. After the pre-loading with DCF-DA, cells were treated with 5 µM of compounds, in the presence or absence of antimycin A. The ROS generation was detected for 30 min. The data represent the mean ± SD of at least three independent experiments and six sample replicates. The control samples (basal condition or in the presence of antimycin A) versus the other samples (in the presence of the various compounds) were compared by the t-Student test; * *p* < 0.05.

In Figure 7, it is clearly shown that the fluorescence intensity markedly increased in antimycin A-treated cells (green bar, Figure 7) in comparison to untreated ones (brown bar, Figure 7). CAPE confirmed its well-known antioxidant activity by strongly reducing the oxidation rate of DCF, both in the presence and absence of antimycin A, while pioglitazone did not show any effect on the ROS generation rate, as it was not significantly different from the control samples. Hybrid compounds greatly reduced intracellular ROS production, both in the control sample and in the presence of antimycin A. All the active compounds (**2**, **3** and CAPE) almost abolish the effect of antimycin A stimulus, decreasing the fluorescence intensity to that of untreated samples. Among the tested compounds, compound **2** displays the highest antioxidant potency, similar to that of CAPE. Noteworthy, the ferulic and caffeic tails attached to the pioglitazone head have converted this inactive pharmacophore in remarkable antioxidant agents.

## 3. Materials and Methods

### 3.1. Chemistry

Chemical reagents were purchased from Merck, TCI and Fluorochem. Pioglitazone was purchased from Merck. CAPE was synthesized according to procedure reported in [45]. All reactions were performed with dry glassware under a nitrogen atmosphere unless otherwise noted. Melting points were measured in glass capillary tubes on a Büchi SMP-20 apparatus and are uncorrected. Nuclear magnetic resonance spectra (NMR) were recorded at 400 MHz for ^1^H and 100 MHz for ^13^C on Varian VXR 400 spectrometer in CDCl_3_, DMSO-*d_6_* or CD_3_OD as solvents. Chemical shifts (*δ*) are given in ppm from tetramethylsilane (TMS) with the solvent resonance as internal standard (CDCl_3_: *δ* 7.26, DMSO-*d_6_*: *δ* 2.50, CD_3_OD: *δ* 3.31 for ^1^H NMR and CDCl_3_: *δ* 77.16, DMSO-*d_6_*: *δ* 39.52, CD_3_OD: *δ* 49.00 for ^13^C NMR). For ^1^H NMR, data are reported as follows: chemical shift, multiplicity (s = singlet, d = doublet, dd = double of doublets, t = triplet, q = quartet, m = multiplet, p = pentet, dt = doublet of triplets, td = triplet of doublets, tt = triplet of triplets, qd = quartet of doublets, br s = broad singlet), coupling constants (Hz) and integration. Monodimensional NOE spectra were acquired using sequence NOESY1D irradiating band of 50 Hz and using mixing time of 2 sec. Chromatographic separations were performed on silica gel columns through flash or gravity column (Kieselgel 40, 0.040–0.063 mm; Merck) chromatography. Reactions were followed by thin-layer chromatography (TLC) on Merck (0.25 mm) glass-packed pre-coated silica gel plates (60 F^254^) that were visualized in an iodine chamber, or with a UV lamp, KMnO_4_, or bromocresol green. All the names were attributed by Chem BioDraw Ultra 22.2.0. Final compounds mass spectra were recorded on a Waters ACQUITY ARC UHPLC/MS system. All final compounds were pure >95% as determined via UHPLC-MS analyses run on a Waters ACQUITY ARC UHPLC/MS system, consisting of a QDa mass spectrometer equipped with an electrospray ionization interface and a 2489 UV/Vis detector. The detected wavelengths (λ) were 254 nm and 365 nm. The analyses were performed on an XBridge BEH C18 column (10 mm × 2.1 mm i.d., particle size 2.5 μm) with an XBridge BEH C18 VanGuard Cartridge precolumn (5 mm × 2.1 mm i.d., particle size 1.8 μm). The mobile phases were H_2_O (0.1% formic acid) (A) and MeCN (0.1% formic acid) (B). Electrospray ionization in positive and negative modes was applied in the mass scan range 50−1200 Da. Method and gradients used were the following: Generic method. Linear gradient: 0−0.78 min, 20% B; 0.78–2.87 min, 20−95% B; 2.87–3.54 min, 95% B; 3.54–3.65 min, 95–20% B; 3.65–5.73, 20% B. Flow rate: 0.8 mL/min.

#### 3.1.1. (*E*)-4-Methoxy-4-oxobut-2-enoic Acid (**6**)

To a solution of dimethyl fumarate (1 g, 6.94 mmol) in 45 mL of acetone, lithium hydroxide 1N (7 mL) was added dropwise using a dropping funnel. The reaction mixture was left stirring at room temperature for 1 h. To terminate the reaction, 60 mL of HCl 2N were added. Organic phase was separated and the aqueous phase was further extracted with ethyl acetate (3 × 40 mL). Organic phases, once reunited, were dried with anhydrous sodium sulphate, concentrated in vacuo, and the crude obtained was purified via column chromatography using ethyl acetate as mobile phase. Compound **6** was obtained as white solid (520 mg, 58%). ^1^H NMR (400 MHz, CD_3_OD) δ 6.48 (s, 2H), 3.49 (s, 3H). ^13^C NMR (100 MHz, CD_3_OD) δ 168.09, 167.26, 135.74, 134.38, 53.06.

#### 3.1.2. (*Z*)-5-(4-Hydroxybenzylidene)thiazolidine-2,4-dione (**7**)

Thiazolidine-2,4-dione (625 mg, 5.12 mmol), piperidine (0.13 mL, 1.28 mmol) and benzoic acid (156 mg, 1.28 mmol) were added to a solution of 4-hydroxybenzaldehyde (600 mg, 5.12 mmol) in 25 mL of toluene. The reaction mixture was left stirring at reflux for 6 h. Once cooled to room temperature, the obtained precipitate was filtered and washed with water and toluene. The precipitate was dried to present **7** as yellow powder (1.04 g, 92%). ^1^H NMR (400 MHz, DMSO-*d_6_*) δ 12.38 (br s, 1H), 10.23 (br s, 1H), 7.70 (s, 1H), 7.45 (d, *J* = 8.4 Hz, 2H), 6.91 (d, *J* = 8.4 Hz, 2H). ^13^C NMR (100 MHz, DMSO-*d_6_*) δ 181.18, 175.80, 159.08, 132.18 (2C), 131.30 (2C), 127.49, 125.51, 117.00.

#### 3.1.3. 5-(4-Hydroxybenzyl)thiazolidine-2,4-dione (**8**)

A total volume of 2.5 mL of cobalt (II) chloride–dimethylglyoxime complex solution (42 mg CoCl_2_·6H_2_O, 250 mg DMG in 5.0 mL DMF) was added to a suspension of **7** (1.51 g, 6.83 mmol) in water (7.26 mL), methanol (5.1 mL) and NaOH 1M (5.79 mL), and this mixture was left stirring at room temperature for 15 min. Sodium borohydride (646 g, 17.08 mmol) under nitrogen atmosphere was added. Subsequently, HCl 6N was added until pH=7 was reached and the reaction mixture was left to be stirred at room temperature for 3 h. HCl 10% was further added to terminate the reaction and the formed precipitate was filtered and washed with water. Once dried, **8** was obtained as white solid (820 mg, 54%). ^1^H NMR (400 MHz, DMSO-*d_6_*) δ 11.97 (br s, 1H), 9.32 (br s, 1H), 7.02 (d, *J* = 8.4 Hz, 2H), 6.68 (d, *J* = 8.4 Hz, 2H), 4.84–4.80 (m, 1H), 3.27–3.23 (m, 1H), 3.02–2.96 (m, 1H). ^13^C NMR (100 MHz, DMSO-*d_6_*) δ 176.63, 172.62, 157.27, 131.14 (2C), 127.66, 116.07 (2C), 54.14, 37.33.

#### 3.1.4. 4-((2,4-Dioxothiazolidin-5-yl)methyl)phenyl Methyl Fumarate (**1**)

To a solution of **6** (100 mg, 0.77 mmol) in 1.2 mL of acetonitrile at 0 °C under nitrogen atmosphere, HOBt (135 mg, 1.00 mmol) and EDC (191 mg, 1.00 mmol) were added. Reaction mixture was left stirring at this temperature for 30 min. After confirming the complete conversion of the starting material in activated complex, intermediate **8** (240 mg, 1.08 mmol) was added and the reaction mixture was left to be stirred overnight at room temperature. After evaporation of the solvent, the crude was purified through column chromatography using DCM: MeOH (9.75:0.25). Compound **1** was obtained as white crystal powder (62 mg, 24%). Mp= 150–151 °C. ^1^H NMR (400 MHz, CDCl_3_) δ 8.32 (br s, 1H), 7.23 (d, *J* = 4.6 Hz, 2H), 7.11 (d, *J* = 4.6 Hz, 2H), 7.01 (s, 2H), 4.51–4.48 (m, 1H), 3.82 (s, 3H), 3.52–3.48 (m, 1H), 3.17–3.11 (m, 1H). ^13^C NMR (100 MHz, CDCl_3_) δ 174.05, 170.17, 165.23, 163.35, 149.86, 135.11, 133.84, 132.96, 130.51 (2C), 121.84 (2C), 53.26, 52.62, 38.10. MS [ESI-] *m*/*z* 334.13 [M-H]^−^.

#### 3.1.5. (*Z*)-5-(4-Nitrobenzylidene)thiazolidine-2,4-dione (**9**)

Thiazolidine-2,4-dione (773 mg, 6.60 mmol), piperidine (0.16 mL, 1.65 mmol) and benzoic acid (201 mg, 1.65 mmol) were added to a solution of 4-nitrobenzaldehyde (1 g, 6.60 mmol) in 37 mL of toluene. The reaction mixture was left stirring at reflux for 6 h. Once cooled to room temperature, the obtained precipitate was filtered and washed with water and toluene. The precipitate was dried, resulting in **9** as yellow powder (1.09 g, 66%). ^1^H NMR (400 MHz, DMSO-*d_6_*) δ 12.78 (br s, 1H), 8.32 (d, *J* = 11.2 Hz, 2H), 7.88 (s, 1H), 7.84 (d, *J* = 11.2 Hz, 2H). ^13^C NMR (100 MHz, DMSO-*d_6_*) δ 167.15, 167.15, 147.59, 139.52, 131.03 (2C), 129.20, 128.18, 124.38 (2C).

#### 3.1.6. 5-(4-Aminobenzyl)thiazolidine-2,4-dione (**10**)

A total volume of 2.50 mL of cobalt (II) chloride–dimethylglyoxime complex solution (42 mg CoCl_2_·6H_2_O, 250 mg DMG in 5.0 mL DMF) was added to a suspension of **9** (1.07 g, 4.26 mmol) in water (4.5 mL), methanol (3.2 mL) and NaOH 1M (3.6 mL), and this mixture was left stirring at room temperature for 15 min. Sodium borohydride (726 mg, 19.19 mmol) under nitrogen atmosphere was added. Subsequently, HCl 6N was added until pH = 7 was reached and the reaction mixture was left stirring at room temperature for 3 h. HCl 10% was further added to terminate the reaction and the formed precipitate was filtered and washed with water. Once dried, **10** was obtained as yellow powder (524 mg, 55%). ^1^H NMR (400 MHz, DMSO-*d_6_*) δ 6.89 (d, *J* = 8 Hz, 2H), 6.51 (d, *J* = 8 Hz, 2H) 4.78–4.76 (m, 1H), 3.21–3.17 (m, 1H), 2.94–2.89 (m, 1H). ^13^C NMR (100 MHz, DMSO-*d_6_*) δ 175.67, 171.72, 146.89, 129.62 (2C), 123.72, 114.06 (2C), 53.41, 36.48.

#### 3.1.7. General Procedure for the Synthesis of Compounds **13**, **14**, **3** and **5**

To a solution of the corresponding acid (**11** or **12**, 1 eq) in DMF (1–2 mL) at 0 °C, HOBt (1.3 eq) and EDC (1.3 eq) were added. Reaction mixture was left stirring at this temperature for 30 min. After confirming the complete conversion of starting material in activated complex, triethylamine (1.3 eq) and **8** or **10** (1.3 eq) were added and the reaction mixture was left stirring for 36 h. After evaporation of the solvent, the crude was purified through column chromatography.

#### 3.1.8. 4-((2,4-Dioxothiazolidin-5-yl)methyl)phenyl (*E*)-3-(3,4-Dimethoxyphenyl)acrylate (**13**)

Compound **13** was synthesized from **11** (460 mg, 2.24 mmol) and **8** (650 mg, 2.91 mmol). Compound was eluted with DCM: MeOH (9.8:0.2), affording **13** as white solid (230 mg, 25%). ^1^H NMR (400 MHz, DMSO-*d_6_*) δ 12.08 (br s, 1H), 7.77 (d, *J* = 15.8 Hz, 1H), 7.44 (s, 1H), 7.33–7.29 (m, 3H), 7.14 (d, *J* = 6.8 Hz, 1H), 7.01 (d, *J* = 8 Hz, 1H), 6.78 (d, *J* = 15.8 Hz, 1H), 4.95–4.92 (m, 1H), 3.81 (s, 3H), 3.80 (s, 3H), 3.41–3.33 (m, 1H), 3.18–3.15 (m, 1H). ^13^C NMR (100 MHz, DMSO-*d_6_*) δ 175.84, 171.74, 165.35, 151.60, 149.81, 149.26, 146.89, 134.44, 130.47 (2C), 126.91, 123.66, 121.96 (2C), 114.72, 111.81, 110.86, 55.89, 55.82, 52.78, 36.73.

#### 3.1.9. (*E*)-3-(3,4-Dimethoxyphenyl)-*N*-(4-((2,4-dioxothiazolidin-5-yl)methyl)phenyl)acrylamide (**14**)

Compound **14** was synthesized from **11** (145 mg, 0.70 mmol) and **10** (201 mg, 0.91 mmol). Compound was eluted with DCM: MeOH (9.8:0.2), affording **14** as yellowish powder (130 mg, 45%). ^1^H NMR (400 MHz, DMSO-*d_6_*) δ 10.09 (br s, 1H), 7.60 (d, *J* = 8.4 Hz, 2H), 7.49 (d, *J* = 15.4 Hz, 1H), 7.17–7.14 (m, 4H), 6.97 (d, *J* = 8.4 Hz, 1H), 6.66 (d, *J* = 15.4, 1H), 4.86–4.83 (m, 1H), 3.79 (s, 3H), 3.76 (s, 3H), 3.33–3.29 (m, 1H), 3.07–3.05 (m, 1H). ^13^C NMR (100 MHz, DMSO-*d_6_*) δ 175.81, 171.71, 163.67, 150.25, 148.79, 140.13, 138.24, 131.37, 129.48 (2C), 127.33, 121.66, 119.67, 118.95 (2C), 111.62, 109.84, 55.44, 55.28, 52.86, 36.59.

#### 3.1.10. 4-((2,4-Dioxothiazolidin-5-yl)methyl)phenyl (*E*)-3-(4-hydroxy-3-methoxyphenyl)acrylate (**3**)

Compound **3** was synthesized from **12** (100 mg, 0.52 mmol) and **8** (151 mg, 0.68 mmol). Compound was eluted with DCM: MeOH (9.9:0.1), which afforded **3** as white crystal powder (21 mg, 10%). Mp= 155–156 °C. ^1^H NMR (400 MHz, DMSO-*d_6_*) δ 12.03 (br s, 1H), 9.67 (br s, 1H), 7.71 (d, *J* = 15.6 Hz, 1H), 7.38 (s, 1H), 7.27 (d, *J* = 8 Hz, 2H), 7.14 (d, *J* = 9 Hz, 1H), 7.11 (d, *J* = 8 Hz, 2H), 6.79 (d, *J* = 9 Hz, 1H), 6.66 (d, *J* = 15.6 Hz, 1H), 4.92–4.89 (m, 1H), 3.80 (s, 3H), 3.40–3.36 (m, 1H), 3.15–3.12 (m, 1H). ^13^C NMR (100 MHz, DMSO-*d_6_*) δ 176.05, 171.97, 165.68, 150.24, 150.06, 148.42, 147.49, 134.62, 130.69 (2C), 125.86, 124.08, 122.21 (2C), 115.99, 113.80, 111.93, 56.17, 53.03, 36.95. MS [ESI+] *m*/*z* 400.35 [M+H]^+^.

#### 3.1.11. (*E*)-*N*-(4-((2,4-Dioxothiazolidin-5-yl)methyl)phenyl)-3-(4-hydroxy-3-methoxyphenyl)acrylamide (**5**)

Compound **5** was synthesized from **12** (350 mg, 1.80 mmol) and **10** (520 mg, 2.34 mmol). Compound was eluted with DCM: MeOH (9.7:0.3), which afforded **5** as yellow orange powder (150 mg, 21%). Mp = 151–152 °C. ^1^H NMR (400 MHz, CD_3_OD) δ 7.62–7.56 (m, 3H), 7.24 (d, *J* = 8.4 Hz, 2H), 7.17 (s, 1H), 7.09 (d, *J* = 8 Hz, 1H), 6.82 (d, *J* = 8 Hz, 1H), 6.61 (d, *J* = 15.6, 1H), 4.74–4.71 (m, 1H), 3.90 (s, 3H), 3.45–3.40 (m, 1H), 3.17–3.11 (m, 1H). ^13^C NMR (100 MHz, CD_3_OD) δ 177.42, 173.46, 167.22, 150.13, 149.32, 143.28, 139.33, 133.56, 130.92 (2C), 128.20, 123.43, 121.21, 119.00, 116.54 (2C), 111.71, 56.40, 54.60, 38.66. MS [ESI-] *m*/*z* 397.04 [M-H]^−^.

#### 3.1.12. General Procedure for the Synthesis of Compounds **2** and **4**

A solution of **13** or **14** (1 eq) in 0.15–0.20 mL of anhydrous DCM was cooled at 0 °C. Boron tribromide 1M in DCM (2.5 eq) was added to the solution and the reaction was left stirring at the same temperature until completion. A total volume of 5 mL of cold water was added to terminate the reaction and the mixture extracted with ethyl acetate (3 × 4 mL). Organic phases, once reunited, were dried with anhydrous sodium sulphate, concentrated in vacuo, and the crude obtained was purified via column chromatography using dichloromethane/methanol as mobile phase.

#### 3.1.13. 4-((2,4-Dioxothiazolidin-5-yl)methyl)phenyl (*E*)-3-(3,4-Dihydroxyphenyl)acrylate (**2**)

Compound **2** was synthesized from **13** (110 mg, 0.27 mmol). Compound was eluted with DCM: MeOH (9.7:0.3), which afforded **2** as white solid (16 mg, 16%). Mp= 150–151 °C. ^1^H NMR (400 MHz, CD_3_OD) δ 7.73 (d, *J* = 16 Hz, 1H), 7.31 (d, *J* = 8 Hz, 2H), 7.12–7.10 (m, 3H), 7.00 (d, *J* = 10 Hz, 1H), 6.80 (d, *J* = 8.4, 1H), 6.45 (d, *J* = 16 Hz, 1H), 4.75–4.71 (m, 1H), 3.50–3.45 (m, 1H), 3.18–3.14 (m, 1H). ^13^C NMR (100 MHz, CD_3_OD) δ 177.87, 173.90, 168.05, 152.15, 150.53, 149.25, 147.40, 135.93, 131.97 (2C), 128.05, 123.88, 123.39 (2C), 117.05, 115.84, 114.63, 54.98, 39.10. MS [ESI+] *m*/*z* 408 [M+Na]^+^. MS [ESI+] *m*/*z* 386.24 [M+H]^+^.

#### 3.1.14. (*E*)-3-(3,4-Dihydroxyphenyl)-*N*-(4-((2,4-dioxothiazolidin-5-yl)methyl)phenyl)acrylamide (**4**)

Compound **4** was synthesized from **14** (88 mg, 0.21 mmol). Compound was eluted with DCM: MeOH (9.5:0.5), which afforded **4** as yellow powder (55 mg, 67%). Mp= 148–149 °C. ^1^H NMR (400 MHz, CD_3_OD) δ 7.56 (d, *J* = 8.6 Hz, 2H), 7.47 (d, *J* = 15.6 Hz, 1H), 7.18 (d, *J* = 8.6 Hz, 2H), 7.02 (s, 1H), 6.91 (d, *J* = 8.5 Hz, 1H), 6.75 (d, *J* = 8.5 Hz, 1H), 6.50 (d, *J* = 15.6 Hz, 1H), 4.69–4.65 (m, 1H), 3.40–3.35 (m, 1H), 3.11–3.05 (m, 1H). ^13^C NMR (100 MHz, CD_3_OD) δ 177.28, 173.36, 167.20, 148.88, 146.61, 143.32, 139.18, 133.37, 130.76 (2C), 128.09, 122.27, 121.09, 118.49, 116.356 (2C), 115.02, 54.47, 38.51. MS [ESI+] *m*/*z* 385.24 [M+H]^+^.

### 3.2. hMAOs Activity Assays

Human recombinant *h*MAO-A and *h*MAO-B expressed in Baculovirus infected BT1 cells (5 mg/mL) and horseradish peroxidases were purchased from Fluka-Sigma-Aldrich s.r.l. (Italy). A Cary Eclipse fluorimeter (Varian Inc., Palo Alto, CA, USA) was used for fluorescence measurements. The stock solutions of compounds and pioglitazone were prepared in dimethyl sulfoxide. The MAO activity assay method was chosen after the evaluation of the possible interferences of the caffeic and ferulic derivatives with two different assay methods. At first, the peroxidase-coupled continuous assay that detect the H_2_O_2_ generation rate was considered. It uses the Amplex Red reagent as the fluorogenic substrate for horseradish peroxidase. By building calibration curves with H_2_O_2_ in the presence of the various compounds (**1**–**5**, up to 20 μM), a strong interference (reduction in the slopes of the calibration curves) was found, as reported also for other phenolic compounds [46]. Consequently, the kynuramine assay, which is based on the fluorimetric detection of the aldehyde (4-hydroxyquinoline) produced by the oxidative deamination of kynuramine (MAO substrate), was evaluated and chosen [47,48]. Indeed, from the calibration curves built using the standard 4-hydroxyquinoline, a slight decrease in their slopes was found only at compounds (**1**–**5**) concentration higher than 20 μM.

By applying this method, *h*MAO activity assays were performed in K/Pi 0.1 M, EDTA 1 mM (total volume: 200 μL; pH 7.4, T = 37 °C), in the presence and in absence of various concentrations of the different compounds (0.2–20 μM and 2–50 μM in the case of *h*MAO–B and *h*MAO-A, respectively). 

MAOs stock solutions were diluted with assay buffer in the presence or absence of the tested compound to obtain a final concentration of 0.005 mg/mL for MAO-A and 0.010 mg/mL for MAO-B; then, kynuramine (10–300 μM final concentration) was added to the assay solution. After 45–60 min of incubation at 37 °C, the reaction was stopped by the addition of 2 M NaOH (80 μL) and 480 μL of distilled water. Kynuramine deaminated by MAO spontaneously cyclizes to provide 4-hydroxyquinoline, the amount of which was determined by the fluorescence intensity of the peak of its emission spectra (λ_exc_ = 330 nm and λ_em_ 330–530 nm). Then, the rate of reaction was calculated using a specific calibration curve (in the presence of the same amount of the tested compound) built with the standard 4-hydroxyquinoline.

To determinate the K_i_ values for the various compounds, the substrate was used at a concentration lower than K_m_ ([S] = 10 μM; K_M_ = 34 ± 8 μM, for both the MAOs). Under these experimental conditions, the Michaelis–Menten equation for rate of reaction, can be simplified as follows: V ≈ (V_max_/K_M_) [E]_0_[S]_0_ [49]. In the presence of an inhibitor, one or both the kinetic parameters, K_M_ and V_max_, might be affected by its presence, depending on the inhibition mechanism. In particular, in the presence of a reversible inhibitor, both the kinetic parameters could be affected, and the apparent kinetic parameter would then become: K_M I_ = K_M,0_ (1 + [I]/K_i_)/(1 + [I]/(αK_i_)), and V_maxI_ = V_max0_/(1 + [I]/(αK_i_)), where α is the factor indicating how much the dissociation constant of the enzyme-substrate-I complex is different from that of the enzyme–I complex (also named K_i_) [49]. Consequently, the ratio of the initial rate of reaction in the absence (V_o_) and in the presence (V_I_) of the tested compound (V_o_/V_I_) can be simplified to the ratio of (V_max_/K_M_)_o_/(V_max_/K_M_)_I_ ≈ (1 + [I]/K_i_). By plotting the ratio (V_o_/V_I_) against the compound concentration, a linear dependence of V_o_/V_I_ on the inhibitor concentration was obtained, and the K_i_ values were calculated from the slope values (1/K_i_) resulting from the linear regression analysis. It is worth noting that the IC_50_ values calculated with the same experimental data (GraphPad 9.0 software, GraphPad Software, San Diego, CA, USA) presented Ki values within the range reported in Table 1 in agreement with the relation between IC_50_ and K_i_ for the competitive and mixed mechanism of inhibition [50].

To study the mechanism of inhibition, the steady-state kinetic parameters (V_max_ and K_m_) of MAO-A and MAO-B were determined by performing experiments at different substrate concentrations and in the absence and presence of different concentrations of the tested compound. V_max_ and K_m_ were calculated by fitting the Michaelis–Menten equation to the experimental data (initial rate of reactions vs. substrate concentrations), with Sigma Plot software, version 9.0 (Jandel Scientific, San Rafael, CA, USA). The mode of inhibition was determined via global fit analysis (GraphPad 9.0 software, GraphPad Software, San Diego, CA, USA) of the initial rate of reaction vs. substrate concentration plots, in the presence and absence of inhibitors, to fit equations for competitive, mixed, non-competitive and uncompetitive inhibition models; the fit presenting the highest r^2^ value was selected for the calculation of inhibition constants. K_i_ values are expressed as mean ± S.D. All experiments were performed at least in triplicate.

*Time-dependent inhibition studies.* The time-dependent inactivation by **2** was performed as previously reported [48]. In detail, recombinant *h*MAO-B was pre-incubated in the K/Pi buffer for periods from 0 to about 20 min, at 37 °C in the absence (control sample) and in the presence of compound **2** (5 and 20 µM). After various pre-incubation times (at least 5 times after mixing of enzyme and inhibitor), the residual enzyme activity was determined: an aliquot of sample was withdrawn and diluted in the assay buffer (50-fold dilution) and then, the substrate was added under a “saturating” condition (Kyn = 300 µM).

### 3.3. Docking Analysis at hMAO-B and Keap1

The crystal structure of human MAO-B and Keap1 was retrieved from the Protein Data Bank (PDB code: 4A79 and 7X4W) and processed in order to remove ligands and water molecules. Hydrogen atoms were added to the protein structure using standard geometries with the MOE programme (2020.09). To minimize contacts between hydrogens, the structure was subjected to Amber99 force-field minimization until the rms (root mean square) of conjugate gradient was <0.1 kcal·mol^−1^·Å^−1^ (1 Å = 0.1 nm), keeping the heavy atoms fixed at their crystallographic positions. Selected compounds were built using MOE by considering the molecules in both oxidized and reduced states; Gasteiger partial charges (MOE Suite) were added [48]. Docking procedure was performed using Dock (Moe suite) triangle matcher with London dG as placement score. The receptor was subjected to induced fit protocol. The binding score was performed for MAO-B interaction using X-Score, an empirical scoring function which estimates the binding affinity of a given protein–ligand complex, including terms accounting for van der Waals interaction, hydrogen bonding, deformation penalty and hydrophobic effect. The estimated binding affinity is expressed as a dissociate constant of the protein–ligand complex in negative logarithm (pKd), in which, for example, a pKd equal to 9 represents a binding affinity in the nanomolar range, while a pKd equal to 6 indicates one in the micromolar range.

### 3.4. Cell Viability in SH-SY5Y Cells

MTT assay, based on the mitochondrial dehydrogenase activity that reduces 3-(4,5-dimethylthiazol-2-yl)-2,5-diphenyl-tetrazolium bromide (MTT, Sigma, St Louis, MO, USA) is one of the standard assays used to measure the cellular metabolic activity as an indicator of cell viability, proliferation and cytotoxicity in a quantitative colorimetric assay. At day 0, SH-SY5Y cells were plated at a density of 2.5 × 10^4^ viable cells per well in 96-well plates. After treatment, according to the experimental setting, cells were exposed to an MTT solution in serum-free medium (1 mg/mL). Following 4 h of incubation with MTT and treatment with SDS for 24 h, cell viability reduction was quantified by using a Synergy HT multidetection microplate reader (Bio-Tek).

### 3.5. Nrf2 Nuclear Translocation

SH-SY5Y cells (5 × 106) were seeded in 100 mm^2^ dishes and treated for 3 h with 5 μM compounds; afterward, the medium was removed, and cells were washed twice with ice-cold PBS. Cells were subsequently homogenized 15 times using a glass−glass Dounce homogenizer in 0.32 M sucrose, buffered with 20 mM Tris- HCl (pH 7.4), containing 2 mM EDTA, 0.5 mM EGTA, 50 mM β-mercaptoethanol and 20 μg/mL leupeptin, apotrinin and pepstatin. The homogenate was centrifuged at 300× *g* for 5 min to obtain the nuclear fraction. An aliquot of the nuclear fraction was used for protein assay via the Bradford method, whereas the remaining was boiled for 5 min after dilution with sample buffer and subjected to polyacrylamide gel electrophoresis and immunoblotting, as described.

### 3.6. Measurement of Intracellular ROS Generation Rate

HepG2 cells (human hepatocellular carcinoma) were purchased from the American Type Culture Collection (ATCC HB-8065). Cells were grown in MEM (M0894, Merck Life Sciences S.r.l., Milan, Italy) supplemented with 2.2 g/L NaHCO_3_ (S5761, Merck Life Sciences S.r.l., Milan, Italy), 100 U/mL penicillin, 100 μg/mL streptomycin and 0.25 μg/mL amphotericin B (A5955, Merck Life Sciences S.r.l., Milan, Italy) and 10% foetal calf serum (F7524, Merck Life Sciences S.r.l., Milan, Italy). Cells were cultured at 37 °C in a humidified atmosphere incubator containing 5% carbon dioxide in air.

The intracellular production of ROS was detected via a fluorometric method using 2′,7′-dichlorofluorescein diacetate (D6883, Merck Life Sciences S.r.l., Milan, Italy) as probe. HepG2 cells (6 × 10^3^/well) were seeded into a 96-well culture plate in 200 µL of a complete growth medium and incubated for 48 h in standard conditions. The medium was then removed, and cells were washed with 5 mM of PBS–glucose. Then, cells were loaded with 10 μM of 2′,7′-dichlorofluorescein diacetate in 5 mM of PBS–glucose at 37 °C in the dark for 20 min, washed with 5 mM of PBS–glucose, treated with the test compounds at indicated concentrations in 5 mM of PBS–glucose and then analysed. Fluorescence intensity was recorded for 30 min at λ_emx_ = 485 nm and λ_em_ = 527 nm, using a Victor X3 Multilabel plate reader (Perkin Elmer). The rate of ROS generation was calculated relative to untreated cells (controls) after subtraction of autofluorescence value. Results were presented as the mean values of at least three independent experiments performed with six replicates.

## 4. Conclusions

Neurodegenerative disorders represent one of the most important unmet medical needs, which remain not properly addressed due to the complex and not well-defined etiopathology. In this work, we started from the promising properties of pioglitazone, a marketed drug for diabetes, and repositioned it as an MAO-B selective competitive inhibitor, endowed with remarkable neuroprotective properties and able to penetrate the BBB barrier. Although all clinic trials involving pioglitazone for the treatment of neurodegenerative disorders failed [11,12,13], this biological portfolio represented a bright starting point for further chemical tuning. Herein, we tried to enrich pioglitazone pharmacophore with direct and indirect antioxidant properties with the aim to amplify its neuroprotective profile. To this aim, the 5-benzyl-2,4-thiazolidinedione moiety of pioglitazone, acting as a driving motif for MAO-B inhibition, was conjugated with polyphenolic and electrophilic features, which could represent a source for scavenger action and Nrf2 activation, respectively. Firstly, the exploited conjugating approach allowed us to maintain the pioglitazone profile, showing promising reversible *h*MAO-B inhibition and selectivity over the *h*MAO-A isoform, with compound **2** emerging as the best of the series. The same compound, not cytotoxic up to 50 µM, at 5 µM concentration, strongly induced Nrf2 translocation into the nucleus in SHSY-5Y neuroblastoma cells, and significantly reduced cellular ROS content both in physiological and stressed conditions in HepG2 cells. Although a deeper investigation is required to fully depict the neuroprotective properties as well as the exact Nrf2 mechanism of activation, the applied hybridization strategy has proven to be successful. Particularly, by adhering polyphenolic fragments at the pioglitazone head, we have empowered it with Nrf2 and antioxidant properties while almost maintaining its original MAO-B profile. Collectively, the preliminary results achieved with compound **2** can foster the reconsideration about the therapeutic potential of pioglitazone-based compounds for neurodegenerative disorders and hopefully boost future pioglitazone-driven drug discovery campaigns.

## Figures and Tables

**Table 1:** *h*MAO inhibitory activity and selectivity for compounds **1**–**5** with pioglitazone and CAPE as reference compounds.

Cpd.	*h*MAO-B K_i_ (µM)	*h*MAO-A K_i_ (µM)	SI ^a^
Pioglitazone	0.061 ± 0.018	>>100	>>1000
CAPE	1.8 ± 0.2	12 ± 2	6.7
**1**	3.14 ± 0.20	25 ± 4	8.0
**2**	0.53 ± 0.17	35 ± 4	66
**3**	0.99 ± 0.15	32 ± 2	32.3
**4**	2.5 ± 1.0	39 ± 5	15.6
**5**	3.3 ± 1.0	47 ± 4	14.2

^a^ SI stands for selectivity index, calculated as *h*MAO-A K_i_/*h*MAO-B K_i_. All experiments were performed at least in triplicate.

## Data Availability

The data presented in this study are available on request from the corresponding authors.

## Supplementary Materials

The following supporting information can be downloaded at: https://www.mdpi.com/article/10.3390/molecules28217424/s1. NOESY analyses, chemical stability determination and docking poses in Figures S1–S5, Nrf2 nuclear translocation of CAPE in Figure S6 and NMR spectra of final compounds; Table S1: pKd values for *h*MAO-B docking pose.
