# Supplementary material for: Double Attack to Oxidative Stress in Neurodegenerative Disorders: MAO-B and Nrf2 as Elected Targets
Source: Molecules. 2023 Nov 4;28(21):7424. doi: 10.3390/molecules28217424 (PMC10650714; doi:10.3390/molecules28217424)
Supplement: Supplementary file 1 [file molecules-28-07424-s001.zip › molecules-2640884-supplementary.pdf]

Supporting information

Double attack to oxidative stress in neurodegenerative disorders: MAO-B and Nrf2 as elected targets

Filippo Basagni [1], Maria Luisa Di Paolo [2], Giorgio Cozza [2], Lisa Dalla Via [3,4], Francesca Fagiani [5,6], Cristina Lanni [5], Michela Rosini [1,*] and Anna Minarini [1,*]

[1] Department of Pharmacy and Biotechnology, Alma Mater Studiorum-University of Bologna, 40126 Bologna, Italy; filippo.basagni2@unibo.it (F.B.);

[2] Department of Molecular Medicine, University of Padova, Via G. Colombo 3, 35131, Padova, Italy; marialuisa.dipaolo@unipd.it (M.L.D.P), giorgio.cozza@unipd.it (G.C.);

[3] Department of Pharmaceutical and Pharmacological Sciences, Via F. Marzolo 5, University of Padova, 35131, Padova, Italy; lisa.dallavia@unipd.it (L.D.V.);

[4] Consorzio Interuniversitario Nazionale per la Scienza e Tecnologia dei Materiali (INSTM), 50121, Firenze, Italy;

[5] Department of Drug Sciences (Pharmacology Section), University of Pavia, V.le Taramelli 14, 27100, Pavia, Italy; cristina.lanni@unipv.it (C.L.);

[6] Division of Neuroscience, IRCCS San Raffaele Hospital, Via Olgettina 60, 20132, Milan, Italy; fagiani.francesca@hsr.it (F.F);

Table of contents:

NOESY analyses page S2

Determination of **2**'s chemical stability page S3

Figures and Tables page S4

NMR Spectra for final compounds **1-5** page S7

Corresponding authors: anna.minarini@unibo.it (A.M.), michela.rosini@unibo.it (M.R.).

NOESY analyses

A critical step in these synthetic procedures was represented by the coupling reaction between acidic function of **6**, **11** and **12** and phenol **8** or aniline **10** derivatives. Based on its acidity, the -NH imidic function could partially compete with the hydroxy group of **8** or the amino residue of **10** in coupling with the respective acids. With the aim to fully evaluate the occurred attaching site and therefore confirm the structures of the final compounds, deepen NMR studies were conducted on compounds **1** and **13**, taken as examples. Particularly, nuclear Overhauser effect spectroscopy (NOESY) in one dimension was exploited to highlight interaction between nuclei magnetically active through the space. Irradiating the sample at absorption frequencies specific of interacting nucleus, a responding signal is registered coming from nuclei near in space (usually within 5Å) to the irradiated one (i.e. nuclear Overhauser effect, NOE). For compound **1**, we irradiated protons 5-5' and observed a return signal from vicinal 4-4' but also from olefinic hydrogens 6-6', thus confirming proximity in space and the predicted aromatic oxygen as attaching point (Figure S1a). Analogously, we studied compound **13** by irradiating H-7, and verified a return signal from H-6/6', H-7' and H-10 (Figure S1b). These results corroborate the predicted and intended structure of our compounds.

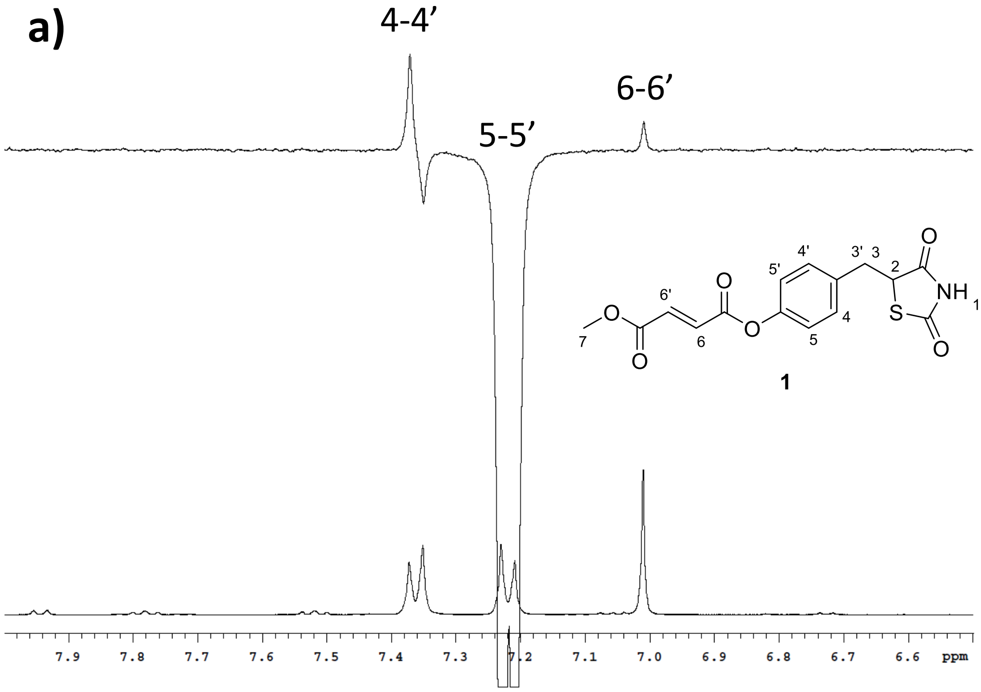

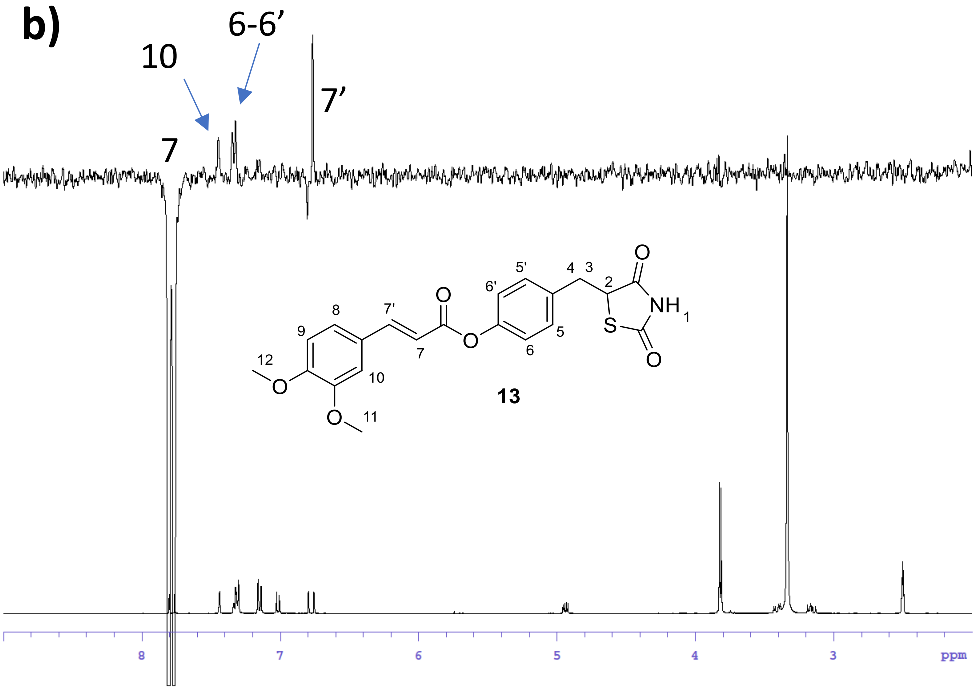

Figure S1. NOE spectra of compounds **1** (a) and **13** (b).

Determination of 2’s chemical stability

Compound **2** was analyzed by UHPLC-MS experiments at t_0 in DMSO stock solution and at $t_{1.5h}$ after incubation in assay medium at 37°C. In the figure below is reported chromatogram registered at 365 nm after 1.5h (same time of *h*MAO assays) incubation in K/Pi 0.1 M buffer, EDTA 1mM (pH 7.4, T=37°C) paired with mass spectra taken from peak compound (Figure 2.21).

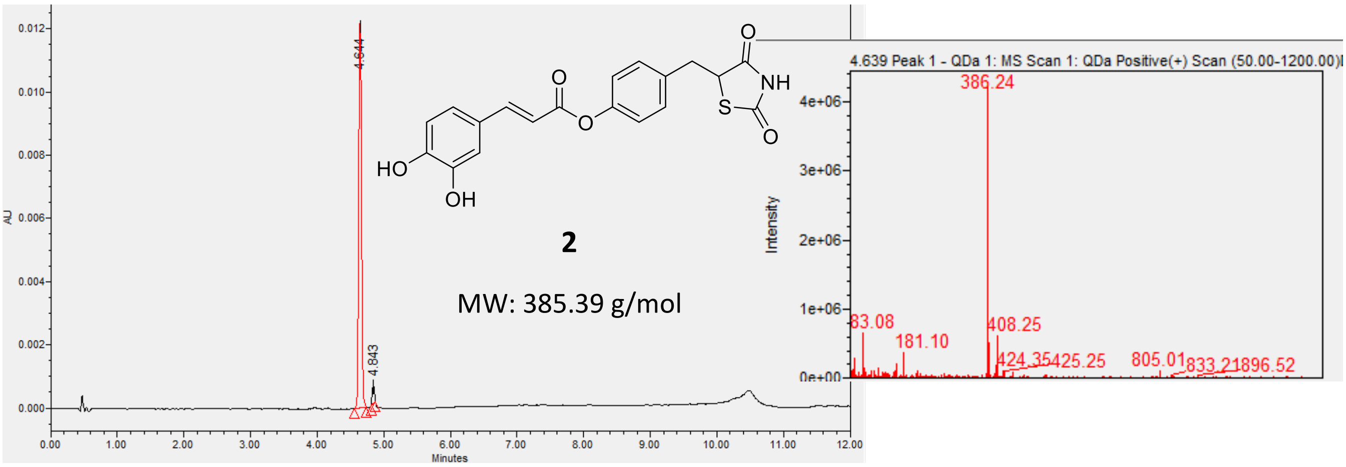

Figure S2. Chromatograms of UHPLC run paired with mass spectra registered at moment of compound elution for compounds **2**.

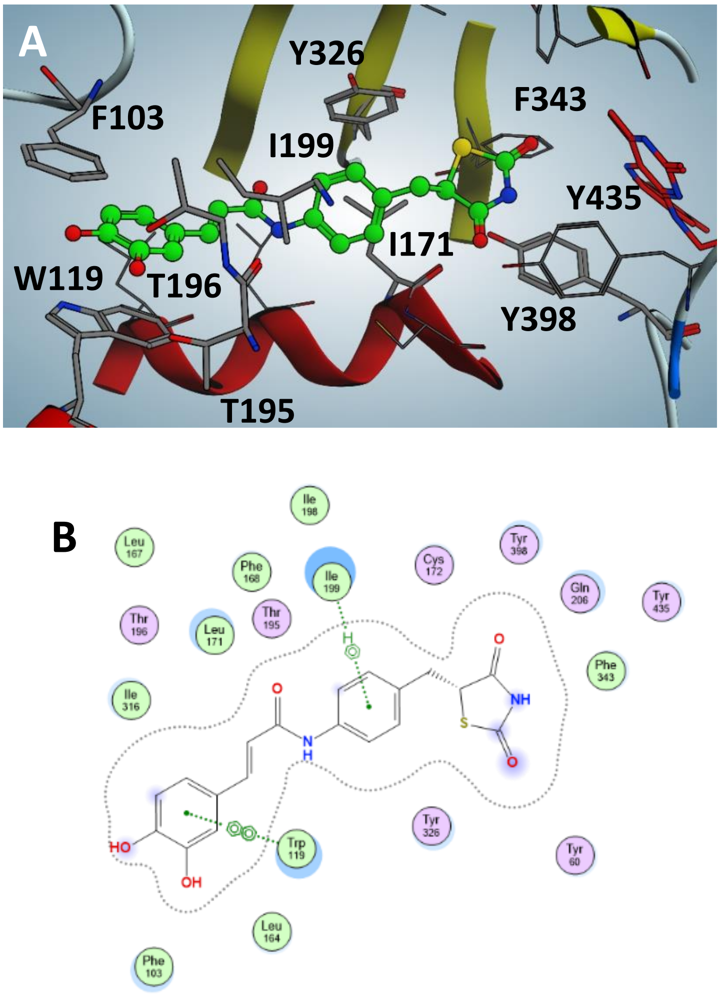

Figure S3. Docking pose of compound **4** against *h*MAO-B with highlighted interactions involved.

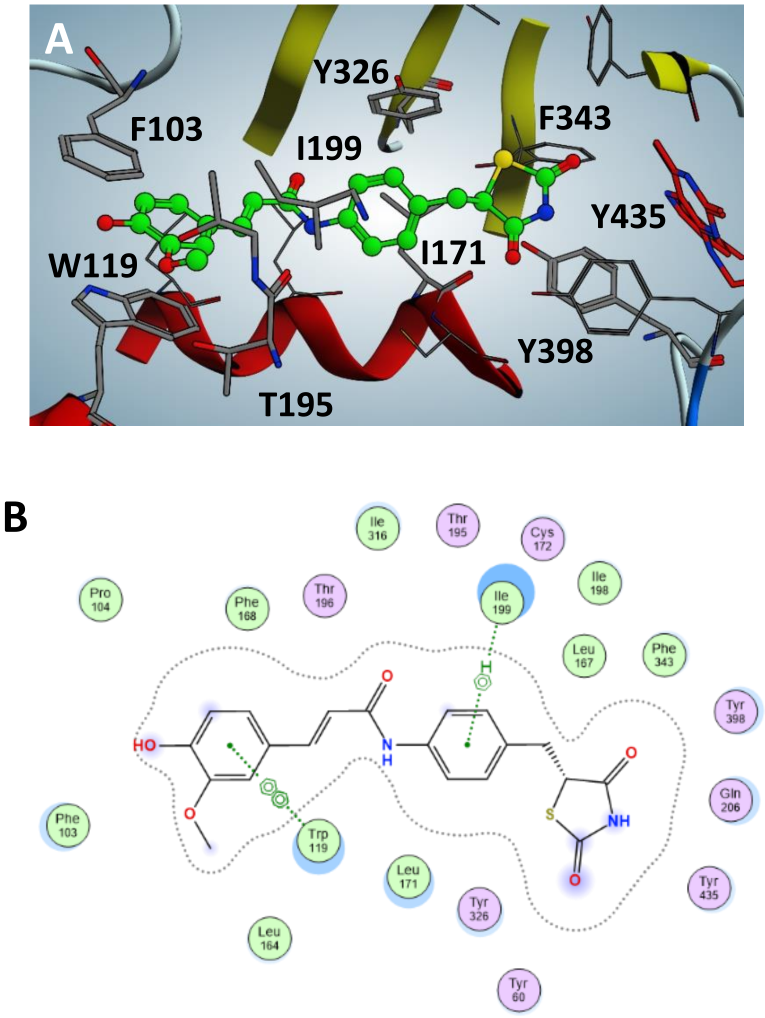

Figure S4. Docking pose of compound **5** against *h*MAO-B with highlighted interactions involved.

Compound	pKd (X-Score)	Significance (vs 2)
**2**	7.2	#
**2 + kynuramine**	5.0	Good
**2_{ox}**	7.0	#
**3**	6.8	#
**4**	6.5	Fair
**5**	6.3	Fair

Table S1: pKd values calculated using X-Score. A fair significance is obtained with pKd differences over 0.5 points (see methods).

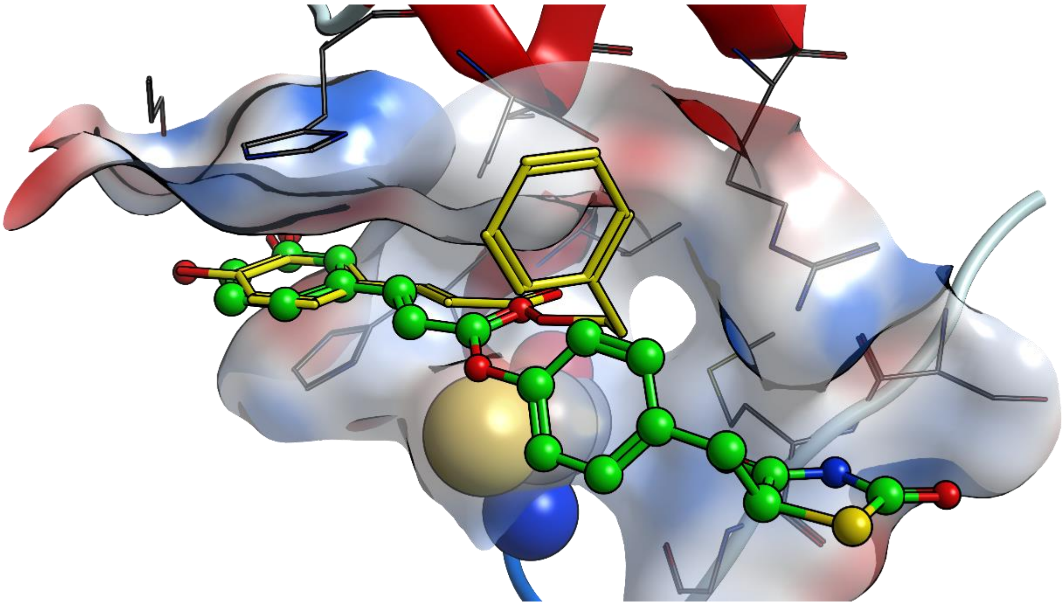

Figure S5. Docking pose of CAPE (yellow) and compound **2** (green) against *h*Keap1 (PDB code: 7X4W). The cavity surface is depicted using the electrostatic Poisson-Boltzmann potential, with blue denoting positively charged regions and red representing negatively charged areas.

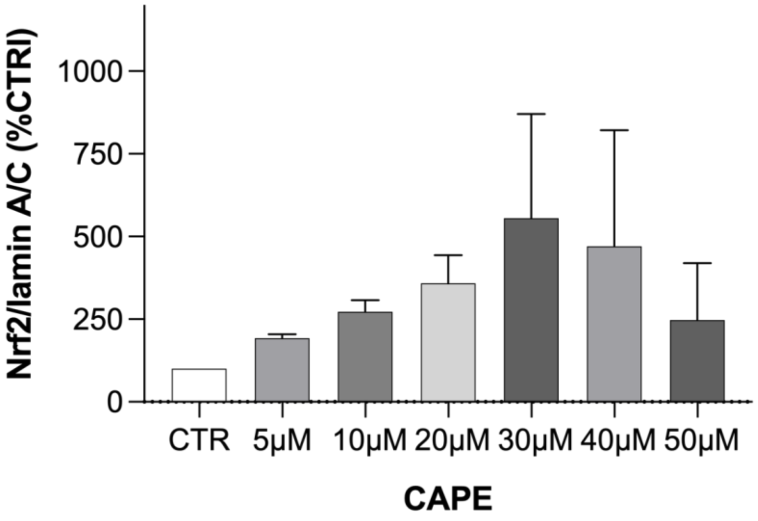

Figure S6. Nrf2 nuclear translocation in SH-SY5Y cells treated with different concentrations of CAPE. SH-SY5Y cells were treated with CAPE 5 µM to 50 µM for 3 hours. After isolation, nuclear extracts were examined by Western blot analysis and Nrf2 expression was determined using an anti-Nrf2 antibody. Anti-lamin A/C was used as protein loading control. Results are shown as Nrf2/Lamin A/C ratio (%CTR) ± SEM.

^{1}H NMR and ^{13}C NMR spectra of compounds 1-5

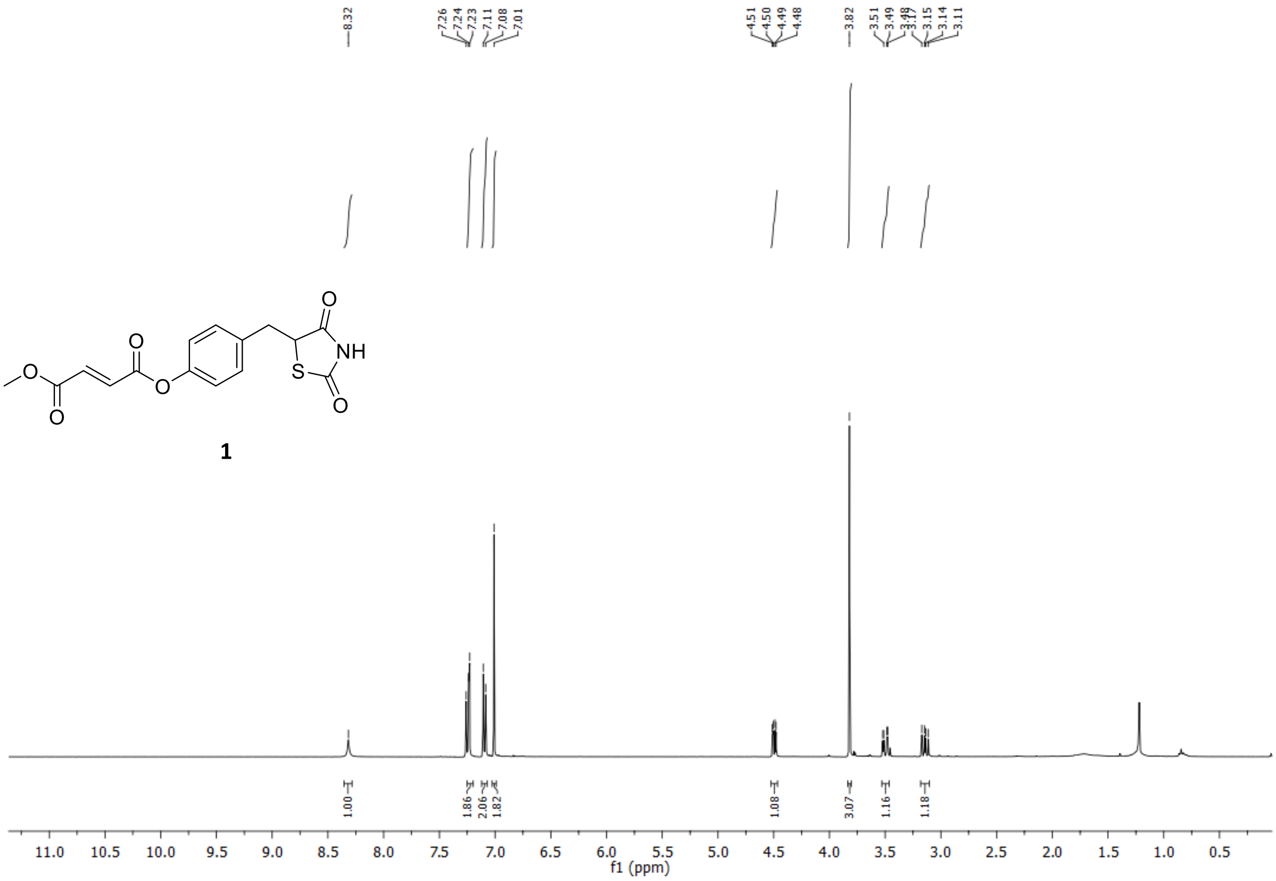

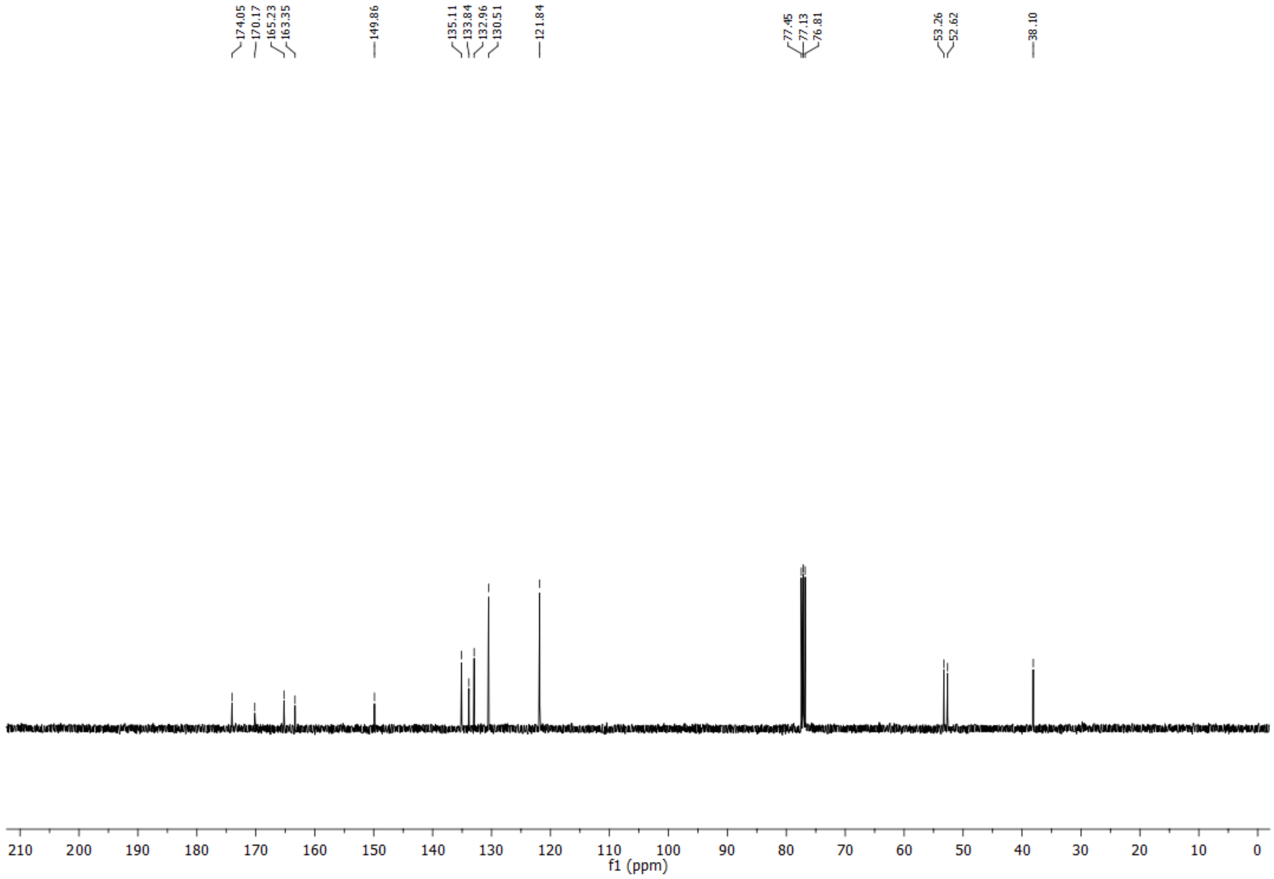

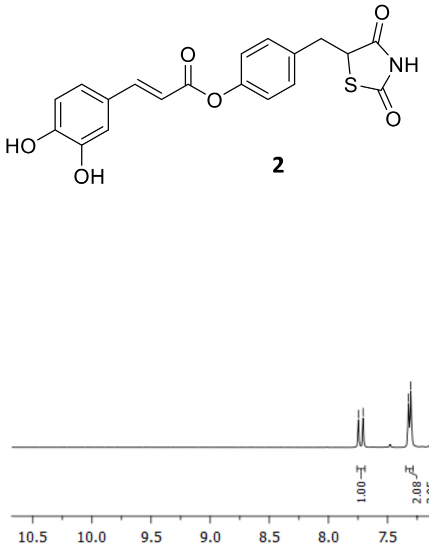
HO
OH
O
O
S
NH
O
O

2

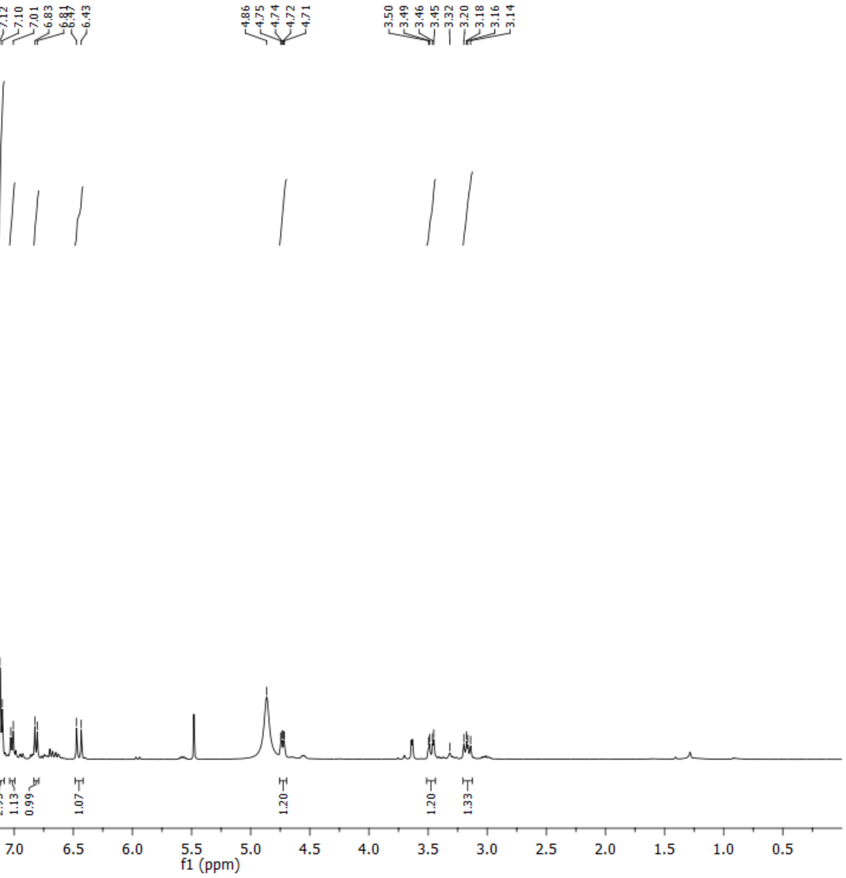
7.75
7.71
7.32
7.30
7.12
7.10
7.01
6.83
6.81
6.47
6.43
4.86
4.75
4.74
4.72
4.71
3.50
3.49
3.46
3.45
3.32
3.20
3.18
3.16
3.14
1.00
2.08
2.95
1.13
0.99
1.07
1.20
1.20
1.33
f1 (ppm)

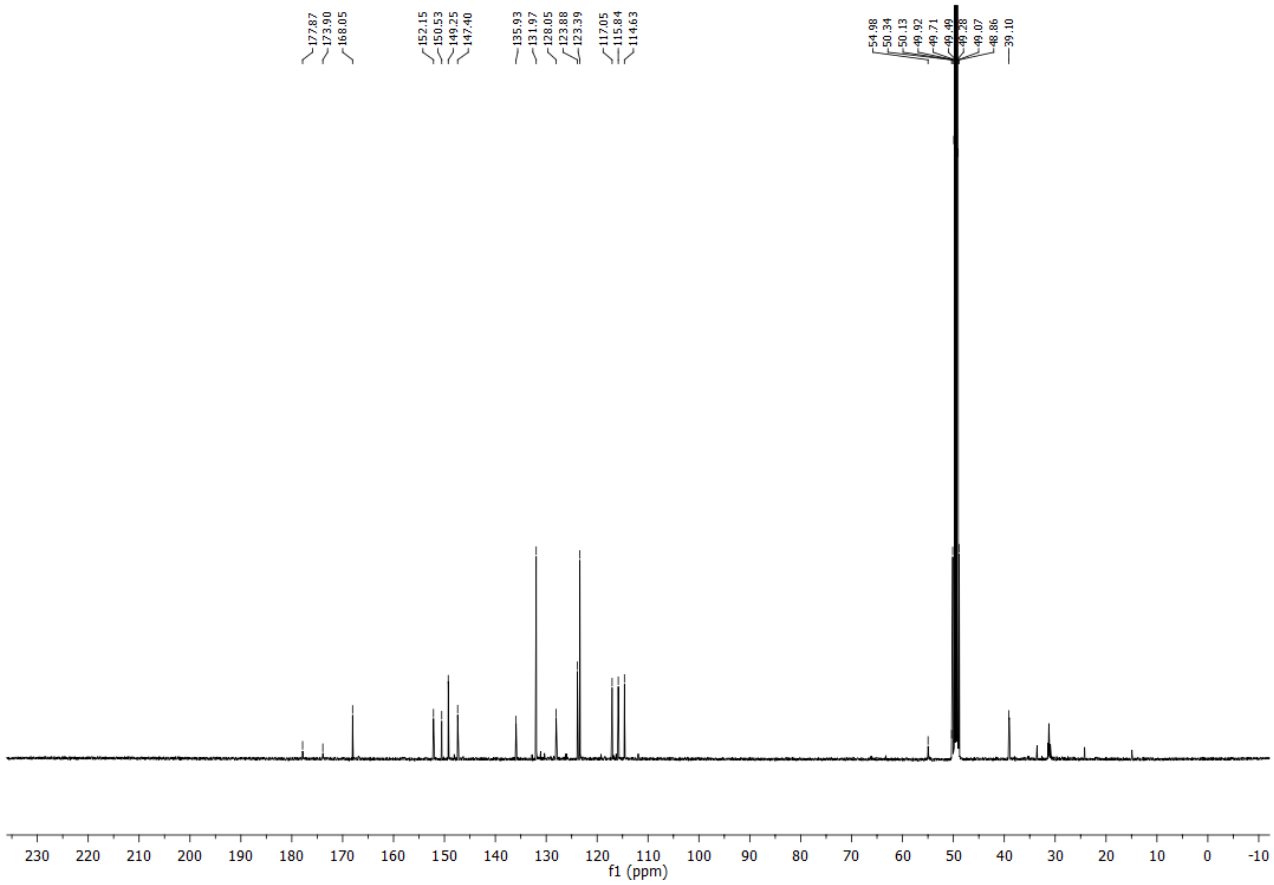
177.87
173.90
168.05
152.15
150.53
149.25
147.40
135.93
131.97
128.05
123.88
123.39
117.05
115.84
114.63
54.98
50.34
50.13
49.92
49.71
49.49
49.28
49.07
48.86
39.10
f1 (ppm)

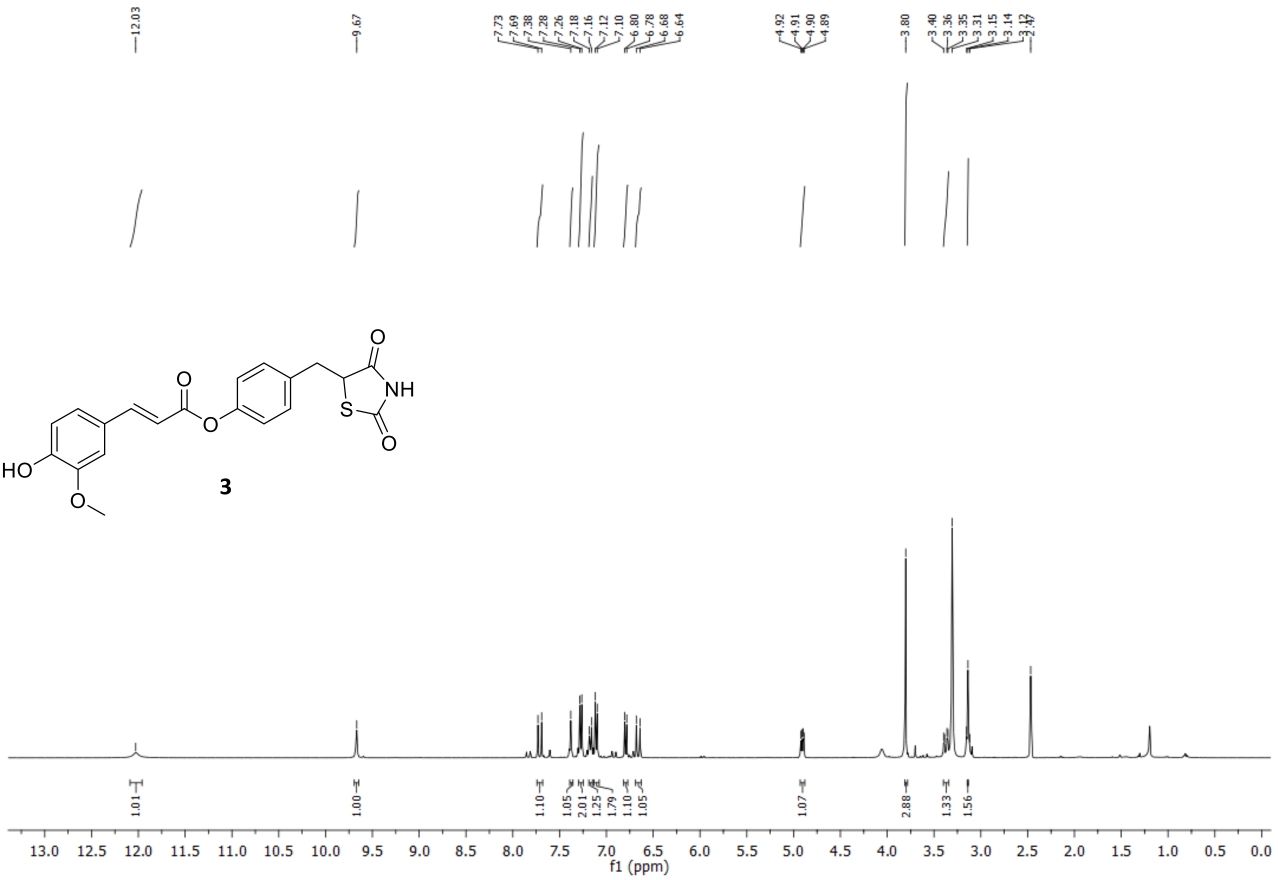
12.03
9.67
7.73 7.69 7.38 7.28 7.26 7.18 7.16 7.12 7.10 6.80 6.78 6.68 6.64
4.92 4.91 4.90 4.89
3.80
3.40 3.36 3.35 3.31 3.15 3.14 3.12 2.47
O
O
NH
S
O
HO
O
3
1.01
1.00
1.10 1.05 2.01 1.25 1.79 1.10 1.05
1.07
2.88
1.33
1.56
13.0 12.5 12.0 11.5 11.0 10.5 10.0 9.5 9.0 8.5 8.0 7.5 7.0 6.5 6.0 5.5 5.0 4.5 4.0 3.5 3.0 2.5 2.0 1.5 1.0 0.5 0.0
f1 (ppm)

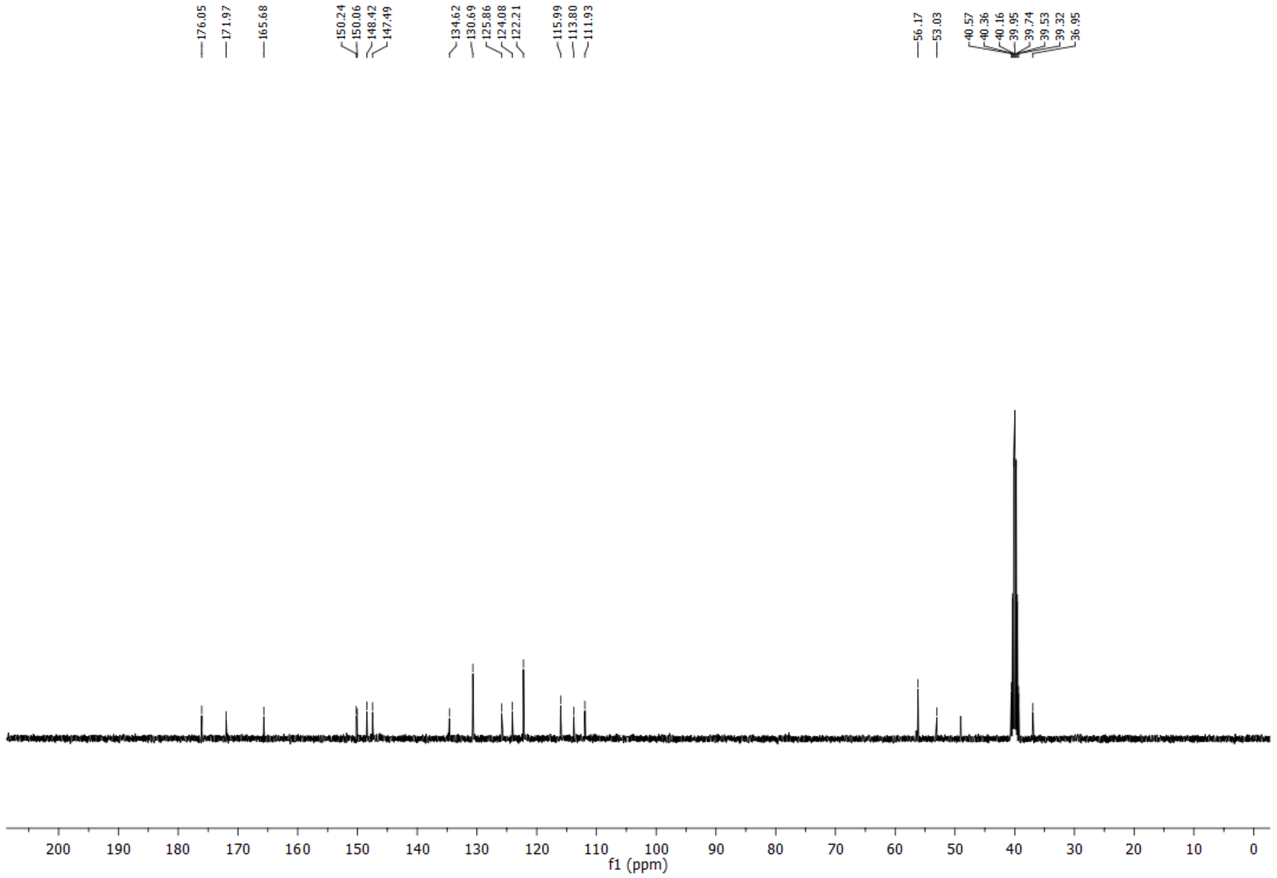
176.05
171.97
165.68
150.24 150.06 148.42 147.49
134.62 130.69 125.86 124.08 122.21
115.99 113.80 111.93
56.17
53.03
40.57 40.36 40.16 39.95 39.74 39.53 39.32 36.95
200 190 180 170 160 150 140 130 120 110 100 90 80 70 60 50 40 30 20 10 0
f1 (ppm)

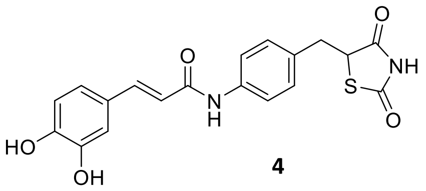
HO
OH
O
N
H
S
NH
O
O

4

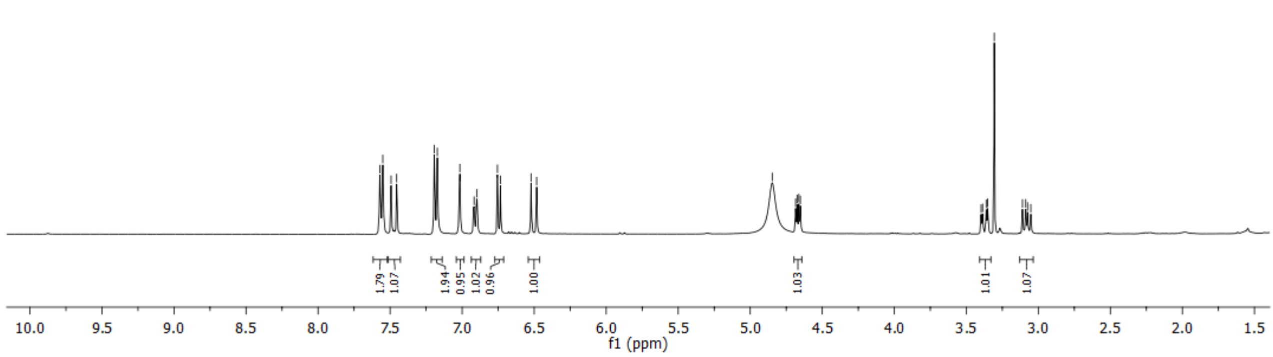
7.57
7.55
7.49
7.45
7.19
7.17
7.02
6.90
6.75
6.73
6.52
6.48
4.85
4.69
4.67
4.66
4.65
3.40
3.39
3.36
3.35
3.31
3.11
3.09
3.07
3.05
1.79
1.07
1.94
0.95
1.02
0.96
1.00
1.03
1.01
1.07
10.0
9.5
9.0
8.5
8.0
7.5
7.0
6.5
6.0
5.5
5.0
4.5
4.0
3.5
3.0
2.5
2.0
1.5
f1 (ppm)

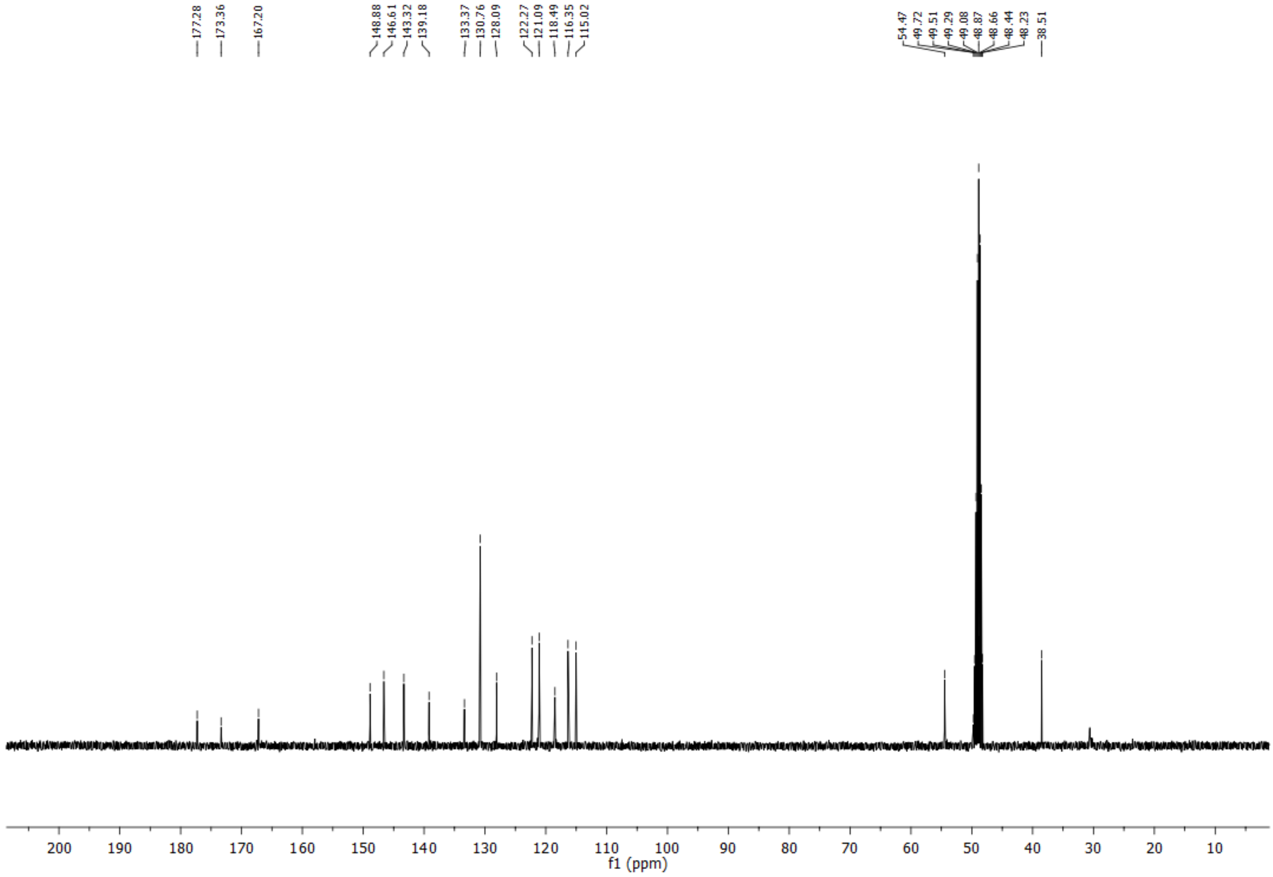
177.28
173.36
167.20
148.88
146.61
143.32
139.18
133.37
130.76
128.09
122.27
121.09
118.49
116.35
115.02
54.47
49.72
49.51
49.29
49.08
48.87
48.66
48.44
48.23
38.51
200
190
180
170
160
150
140
130
120
110
100
90
80
70
60
50
40
30
20
10
f1 (ppm)

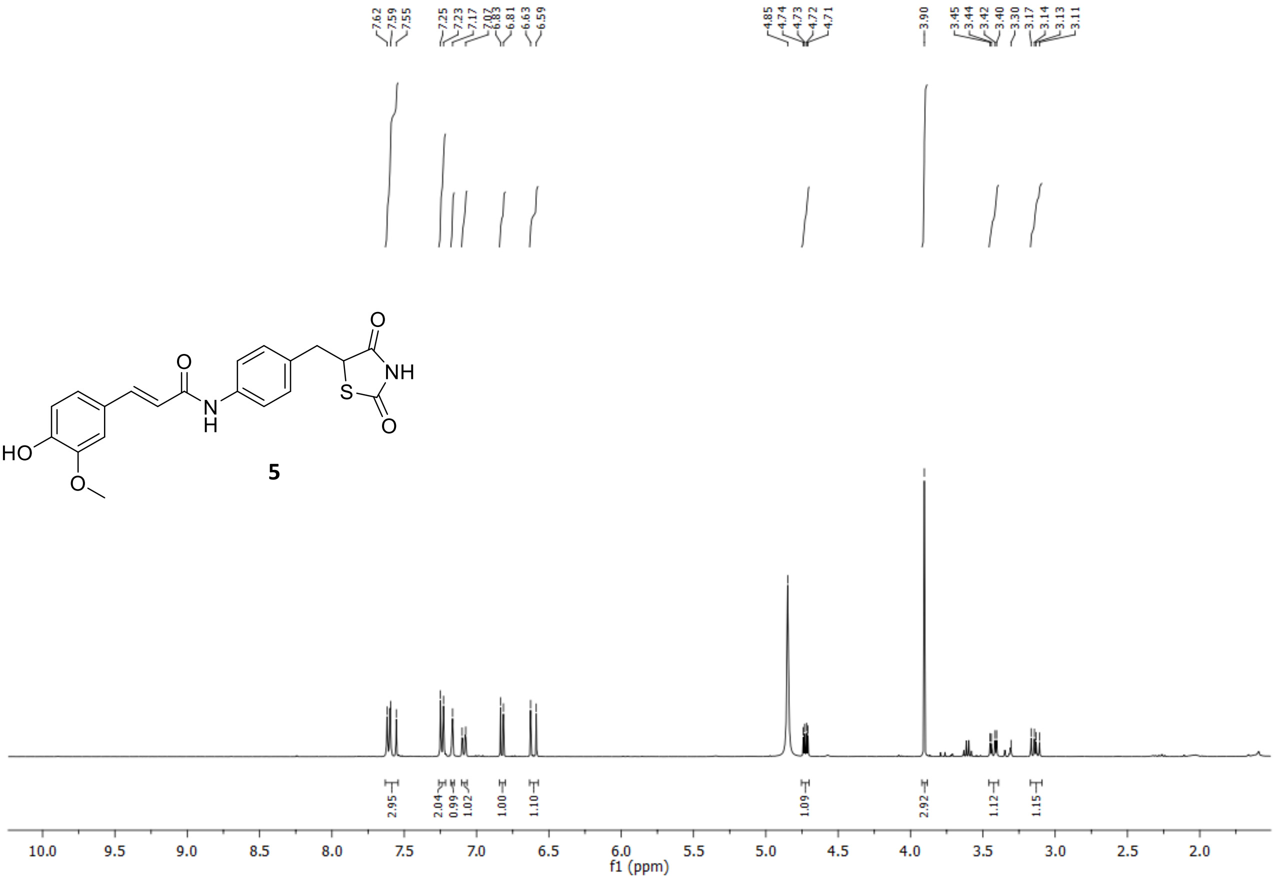

7.62
7.59
7.55
7.25
7.23
7.17
7.07
6.83
6.81
6.63
6.59
4.85
4.74
4.73
4.72
4.71
3.90
3.45
3.44
3.42
3.40
3.30
3.17
3.14
3.13
3.11
O
NH
S
O
O
N
H
HO
O
2.95
2.04
0.99
1.02
1.00
1.10
1.09
2.92
1.12
1.15
10.0 9.5 9.0 8.5 8.0 7.5 7.0 6.5 6.0 5.5 5.0 4.5 4.0 3.5 3.0 2.5 2.0
f1 (ppm)

5

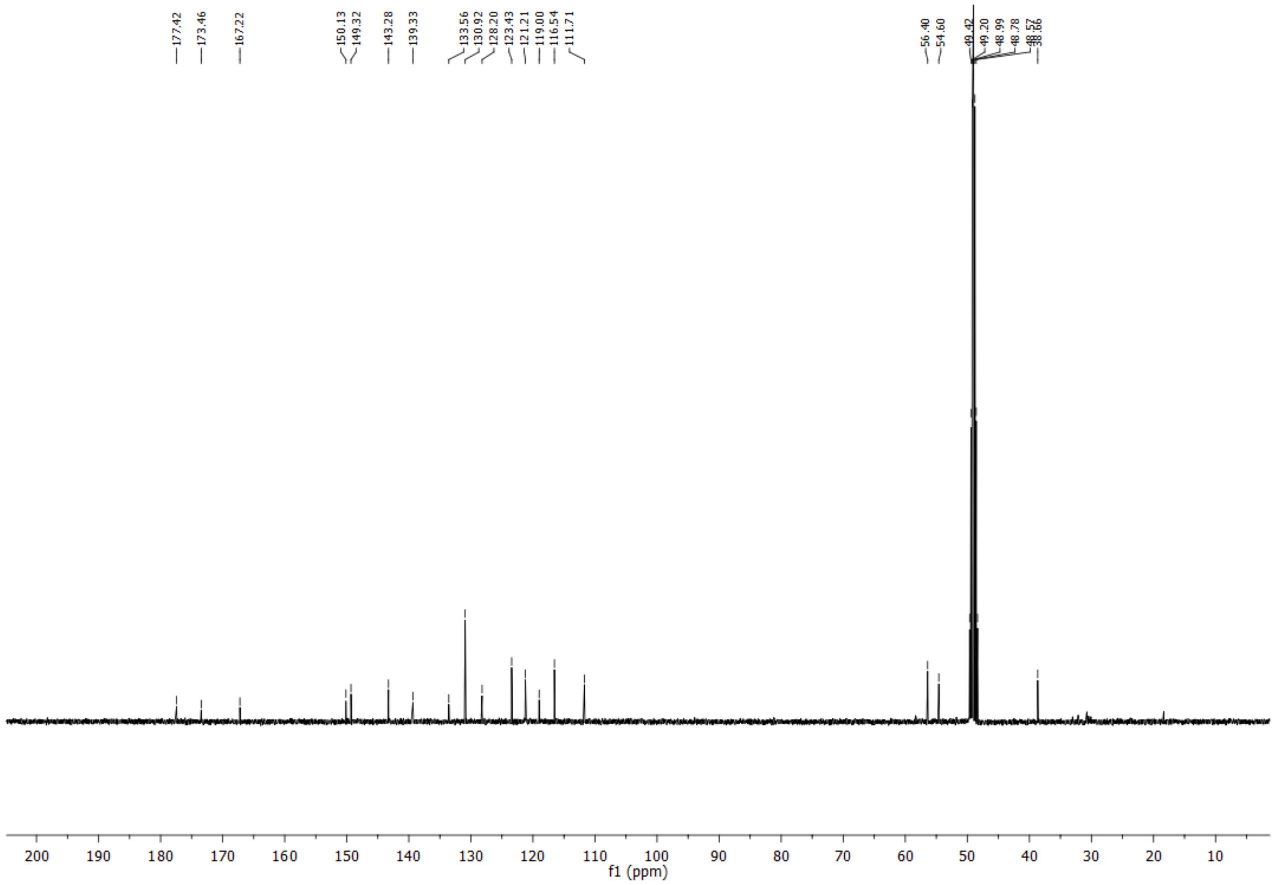

177.42
173.46
167.22
150.13
149.32
143.28
139.33
133.56
130.92
128.20
123.43
121.21
119.00
116.54
111.71
56.40
54.60
49.42
49.20
48.99
48.78
48.57
38.66
200 190 180 170 160 150 140 130 120 110 100 90 80 70 60 50 40 30 20 10
f1 (ppm)
